# Fine-tuning of the pharmacological potential of novel thiazolium ionic liquids by anion alteration†

**DOI:** 10.1039/d1ra07128a

**Published:** 2021-12-22

**Authors:** Mohammad Y. Alfaifi, Ali A. Shati, Serag Eldin I. Elbehairi, Reda F. M. Elshaarawy, Emad M. Gad

**Affiliations:** Biology Department, Faculty of Science, King Khalid University 9004 Abha Saudi Arabia; Cell Culture Lab, Egyptian Organization for Biological Products and Vaccines (VACSERA Holding Company) Giza 12311 Egypt; Chemistry Department, Faculty of Science, Suez University 43533 Suez Egypt; Institut für Anorganische Chemie und Strukturchemie, Heinrich-Heine Universität Düsseldorf Düsseldorf Germany reda.elshaarawy@suezuniv.edu.eg reel-001@hhu.de; Chemistry Department, Faculty of Science, Suez Canal University Ismalia Egypt

## Abstract

A novel series of thiazolium ionic liquids (TILs) bound to chloride (2a–c), tetrafluoroborate (BF_4_) (3a–c), and bis-(trifluoromethanesulfonimide) (Tf_2_N) anions (4a–c) was synthesized and their physicochemical characteristics were investigated using various microanalytical techniques. The pharmacological potential of the new TILs was assessed as chemotherapeutic agents for bacterial infections and ovarian cancer (SKOV-3). Notably, ILs with the same cations become more bactericidal upon their binding with the strongest chaotropic anion (TN_2_f). The *in vitro* toxicity of the TILs toward ovarian carcinoma cell lines (SKOV-3) and normal human skin fibroblast cells (HSF) revealed that all tested TILs have the capacity to induce a dose- and time-dependent decline in SKOV-3 cell viability, with Tf_2_N-linked TILs (4a–c) having a preferable efficacy. In addition, the new compounds showed excellent selectivity for cancer cells (SKOV-3) over healthy cells (HSF). [^i^PBzTh][Tf_2_N] (4b) is the most cytotoxic and specific one and may act as a promising anti-ovarian cancer agent.

## Abstract 2

A novel series of thiazolium ionic liquids (TILs) bound to chloride (2a–c), tetrafluoroborate (BF_4_) (3a–c), and bis-(trifluoromethanesulfonimide) (Tf_2_N) anions (4a–c) was synthesized and their physicochemical characteristics were investigated.

## Graphical abstract

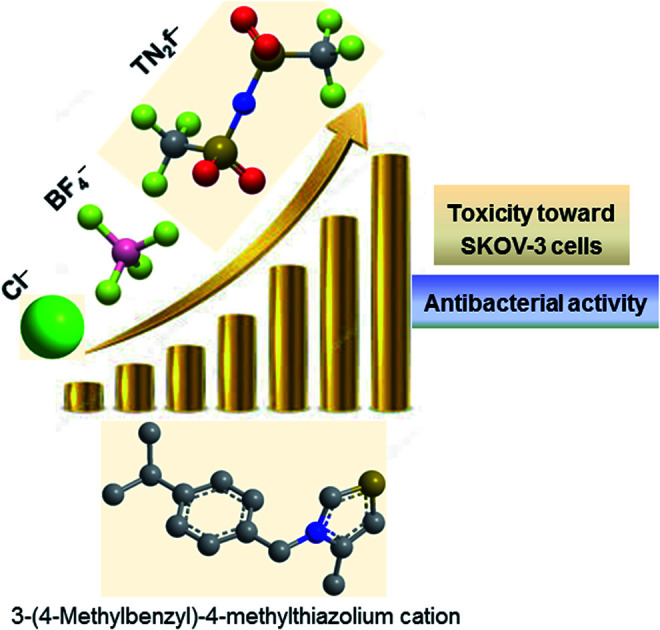

## 1. Introduction

Ovarian cancer (OC) is one of the main death causes among females and the 3^rd^ most prevalent gynecological cancer in developing countries.^1^ Cytoreductive surgery followed by adjuvant chemotherapy (platinum-paclitaxel therapy cocktail) is the most common protocol used in OC therapy.^2^ Chemotherapy was used prior to surgery to reduce tumor size in order to facilitate surgery. However, after surgery, the role of chemotherapy is to eliminate any remaining tumor cells.^2^ As many as 75% of cancer patients initially respond to chemotherapeutic treatment, but most of them relapse due to intrinsic drug resistance and die.^3^ Besides, traditional chemotherapy exhibits a number of disadvantages, including the adverse effects observed when using large dosages, the inability to be absorbed into aqueous media, and a short circulation half-life in the bloodstream.^4,5^

Meanwhile, antimicrobial resistance (AMR) is of great concern nowadays. It is expected that some of the currently utilized drugs will no longer be useful in the future due to their limited efficiencies and side impacts. As a matter of fact, there are even lists established for some AMR microorganisms.^6^ Despite the variety of therapeutic techniques used to prevent this resistance, such as rational medication usage and decreased antibiotic abuse, it is vital to tackle resistance through a variety of strategies, including the production of new bioactive compounds.^6,7^ This is why it is important to continuously seek new, highly effective chemotherapeutic treatments with minimal side effects.

The amazing physical and chemical properties of ionic liquids (ILs), such as excellent thermal and chemical stability, non-flammability, non-volatile, amphiphilic nature, reusability, and electric conductivity,^8^ make them promising candidates for many applications.^9^ In this scenario, they have been efficiently used as solvents and/or catalysts for organic reactions,^10,11^ fabrication of nanomaterials,^12^ electrochemical sensors and biosensors,^13^ optical chemosensors,^14,15^ pH nano-fluorosensing,^16^ scavenging of pollutants from aqueous effluents,^17,18^*etc.*^9^ Furthermore, ILs have been implicated in a variety of biological and pharmaceutical applications due to their low toxicity toward many biological targets and their exceptional pharmacological capabilities.^19^ In addition, the physicochemical and biological characteristics of ILs could be fine-tuned using the numerous reported potential protocols for modifying cations and anions, the main components of ILs.^9^ Noteworthy, tagging of active pharmaceutical ingredients (APIs) by ionic liquids, helps in improving their pharmaceutical properties.^9^ Several reports have documented the effective application of ILs as chemotherapeutic agents.^20^ Despite the many studies that have been reported on the synthesis and applications of ILs, however, most of these reports have been focused only on the common cations (ammonium, imidazolium, pyridinium, or phosphonium and their derivatives).^9,20^ Therefore, exploring other cations is imperative to get more diversified smart ILs for specific-task applications.

The broad-spectrum biochemical and pharmacological properties of the thiazole ring, have made it a popular choice as an active nucleus to design novel chemotherapeutic agents. As a result, its derivatives have been effectively applied in many pharmacological fields, such as antimicrobial, antitumor, antioxidant, antitubercular, anticonvulsant, anti-inflammatory, diuretic, and neuroprotective agents.^21^ In addition, the thiazole ring acts as the main active ingredient in many promising biomolecules, pro-drugs, and clinical drugs.^14^ This motivated us to fabricate novel room-temperature thiazolium ILs for chemotherapeutic applications. To the best of our knowledge, very few efforts have been made by researchers to date to design and fabricate thiazolium ionic liquids. Davis and Forrester have reported the first series of thiazolium ILs for application as catalysts in the benzoin condensation reaction.^22^ Subsequently, a few studies have been performed to explore the potential of other new thiazolium ILs as catalysts,^23–25^ anion exchangers,^26^ and gas fractionators.^27^ Therefore, the current work will be the first study to fine-tune the pharmacological activity of thiazolium ILs by modification of thiazolium cation and its bound anion.

In continuation of our exploring trip for searching of novel multifunctional chemotherapeutic agents,^17,28^ the aforesaid facts motivated us to synthesize a new series of thiazolium ionic liquids (TILs) bound to various *N*-aralkyl side chains and different anions. The newly synthesized TILs were structurally characterized based on their spectral characteristics. The pharmacological potentials of new thiazolium derivatives were *in vitro* investigated against two pathogenic bacterial strains as well as ovarian cancer cell lines.

## 2. Experimental part

### 2.1. Materials and instrumentation

Solvents and chemicals utilized in this study, as well as their suppliers, are listed in the ESI.† Also, the chloromethylation protocol that was used in the preparation of benzyl derivatives was shown in detail in the ESI.† In addition, the structural characterization methods used for the obtained starting materials and ionic liquids have been described in the ESI.†

### 2.2. Synthesis of thiazolium ionic liquids (TILs): 3-aralkyl-4-methylthiazol-3-ium chloride

The following procedure was followed for the preparation of quaternary thiazolium chlorides: a solution of benzyl chloride derivative (1.2 eq.) in 10 mL of dry toluene was gradually added to a solution of 4-methylthiazole (4-MeTh) (1 eq.) in the same solvent under vigorous stirring at room temperature. After the addition was completed, the temperature of the reaction mixture was raised to 80 °C and kept overnight under stirring and reflux at this temperature. After allowing the reaction to cool to ambient temperature and settling the desired products, the soluble unreacted materials are removed by filtration or decantation from the solid or semi-solid products. The obtained products were thoroughly washed with dry toluene (3 × 10 mL), a mixture of diethyl ether-ethyl acetate (3 × 10 mL), and eventually diethyl ether (3 × 10 mL) for the complete removal of any impurities. The solid and semi-solid thiazolium chloride products (2a–c) were dried overnight under a vacuum and have been used for the following reactions without any more purification.

#### 2.2.1 3-(4-Methylbenzyl)-4-methylthiazol-3-ium chloride [MBzTh][Cl] (2a)

Yield: 72%. ^1^H NMR (DMSO-*d*_6_, 300 MHz) *δ* (ppm): 9.97 (s, 1H, Th–H̲), 7.85 (d, *J* = 7.4 Hz, 1H, Ph–H̲), 7.73 (t, *J* = 7.4 Hz, 1H, Ph–H̲), 7.43 (d, *J* = 7.3 Hz, 1H, Ph–H̲), 7.31–7.15 (m, 2H, Th–H̲ and Ph–H̲), 5.39 (s, 2H, Ph–CH̲_2_), 3.79 (s, 3H, Th–CH̲_3_), 2.67 (s, 3H, Ph–CH̲_3_). ^13^C NMR (126 MHz, DMSO-*d*_6_) *δ* (ppm): 157.54 (Th–C̲2), 144.86 (Th–C̲4), 137.71 (Ph–C̲), 129.27 (Ph–C̲), 128.57 (Ph–C̲), 125.68 (Ph–C̲), 117.83 (Th–C̲5), 56.75 (Ph–C̲H_2_), 21.41 (Ph–C̲H_3_), and 14.51 (Th–C̲H_3_). ESI-MS (+ve): 204.09 *m*/*z* [M − Cl^−^, C_11_H_12_NS]^+^; ESI-MS (−ve): 34.97 *m*/*z* [Cl]^−^.

#### 2.2.2 3-(4-Isopropylbenzyl)-4-methylthiazol-3-ium chloride [^i^PBzTh][Cl] (2b)

Yield: 68%. ^1^H NMR (200 MHz, CDCl_3_) *δ* (ppm): 9.96 (s, 1H, Th–H̲), 7.72 (s, br, 3H, Ph–H̲), 7.48 (s, br, 1H, Ph–H̲), 7.30–7.14 (m, 1H, Th–H̲), 5.57 (s, 2H, Ph–CH̲_2_), 3.31 (p, *J* = 1.8 Hz, 1H, CH̲(CH_3_)_2_), 2.86 (s, 3H, Th–CH̲_3_), 1.21 (d, *J* = 6.9 Hz, 6H, CH(CH̲_3_)_2_). ^13^C NMR (125 MHz, CDCl_3_) *δ* (ppm): 160.06 (Th–C̲2), 148.84 (Th–C̲4), 144.71 (Ph–C̲), 129.43 (Ph–C̲), 128.62 (Ph–C̲), 123.44 (Ph–C̲), 120.71 (Th–C̲5), 55.52 (Ph–C̲H_2_), 52.04 (C̲H(CH_3_)_2_), 22.54 (CH(C̲H_3_)_2_), and 14.42 (Th–C̲H_3_). ESI-MS (+ve): 232.22 *m*/*z* [M − Cl^−^, C_14_H_18_NS]^+^; ESI-MS (−ve): 34.97 *m*/*z* [Cl]^−^.

#### 2.2.3 3-(4-(*tert*-Butyl)benzyl)-4-methylthiazol-3-ium chloride [^*t*^BBzTh][Cl] (2c)

Yield: 58%. ^1^H NMR (200 MHz, CDCl_3_) *δ* (ppm): 9.89 (s, 1H, Th–H̲), 7.68–7.59 (m, 3H, Ph–H̲), 7.42 (d, *J* = 7.1 Hz, 1H, Ph–H̲), 7.22 (s, 1H, Th–H̲), 5.49 (s, 2H, Ph–CH̲_2_), 2.79 (s, 3H, Th–CH̲_3_), 1.32 (s, 9H, C̲(CH̲_3_)_3_). ^13^C NMR (126 MHz, DMSO-*d*_6_) *δ* (ppm): 161.46 (Th–C̲2), 148.67 (Th–C̲4), 140.07 (Ph–C̲), 129.24 (Ph–C̲), 126.02 (Ph–C̲), 123.14 (Ph–C̲), 118.44 (Th–C̲5), 55.24 (Ph–C̲H_2_), 35.98 (C̲(CH_3_)_3_), 31.60 (C(C̲H_3_)_3_), and 14.47 (Th–C̲H_3_). ESI-MS (+ve): 246.24 *m*/*z* [M − Cl^−^, C_15_H_20_NS]^+^; ESI-MS (−ve): 34.97 *m*/*z* [Cl]^−^.

#### 2.2.4 Anion metathesis

An 8.46 mmol aliquot of the thiazolium chloride derivative (2a–c) was dissolved in 20 mL of deionized water to obtain a clear solution. Then, while vigorously stirring, a 10.18 mmol aliquot (1.2 eq.) of sodium tetrafluoroborate (NaBF_4_) (1.12 g) or lithium bis-(trifluoromethane-sulfonimide) (LiTf_2_N) (2.92 g) was gradually added to the above solution. Thereafter, the mixture was stirred for 48 hours at room temperature. After leaving the reaction mixture for 15 min to settle, the aqueous layer was discarded and the sticky semi-solid or oily residues were dissolved in dichloromethane, washed with deionized water (5 × 20 mL), and dried overnight over anhydrous magnesium sulfate. Following that, the solvent was evaporated under reduced pressure, and the resulting clear liquid was dried for four days under a high vacuum.

#### 2.2.5 3-(4-Methylbenzyl)-4-methylthiazol-3-ium tetrafluoroborate [MBzTh][BF_4_] (3a)

Yield: 72%. ^1^H NMR (500 MHz, DMSO-*d*_6_) *δ* (ppm): 9.97 (s, 1H, Th–H̲), 7.71 (s, 1H, Ph–H̲), 7.64 (s, 2H, Ph–H̲), 7.32 (s, 1H, Ph–H̲), 7.24 (s, 1H, Th–H̲), 5.32 (s, 2H, Ph–CH̲_2_), 3.88 (s, 3H, Th–CH̲_3_), 2.63 (s, 3H, Ph–CH̲_3_). ^13^C NMR (126 MHz, DMSO-*d*_6_) *δ* (ppm): 157.52 (Th–C̲2), 144.88 (Th–C̲4), 136.86 (Ph–C̲), 129.49 (Ph–C̲), 128.09 (Ph–C̲), 125.69 (Ph–C̲), 117.60 (Th–C̲5), 56.68 (Ph–C̲H_2_), 21.86 (Ph–C̲H_3_), and 14.59 (Th–C̲H_3_). ^11^B NMR (97 MHz, DMSO-*d*_6_) *δ* (ppm): −1.31 (singlet). ^19^F NMR (471 MHz, DMSO-*d*_6_) *δ* (ppm): −148.69 (singlet) (BF̲_4_). ESI-MS (+ve): 204.08 *m*/*z* [M − BF_4_^−^, C_11_H_12_NS]^+^; ESI-MS (−ve): 86.81 *m*/*z* [BF_4_]^−^.

#### 2.2.6 3-(4-Isopropylbenzyl)-4-methylthiazol-3-ium tetrafluoroborate [^i^PBzTh][BF_4_] (3b)

Yield: 65%. ^1^H NMR (200 MHz, CDCl_3_) *δ* (ppm): 10.03 (s, 1H, Th–H̲), 7.74 (d, *J* = 7.1 Hz, 2H, Ph–H̲), 7.67 (d, *J* = 7.1 Hz, 1H, Ph–H̲), 7.60 (d, *J* = 7.2 Hz, 1H, Ph–H̲), 7.48–7.33 (m, 1H, Th–H̲), 5.40 (s, 2H, Ph–CH̲_2_), 3.78 (s, 3H, Th–CH̲_3_), 3.33 (p, *J* = 1.8 Hz, 1H, CH̲(CH_3_)_2_), 1.23 (d, *J* = 6.8 Hz, 6H, CH(CH̲_3_)_2_). ^13^C NMR (125 MHz, CDCl_3_) *δ* (ppm): 160.06 (Th–C̲2), 146.03 (Th–C̲4), 144.71 (Ph–C̲), 132.05 (Ph–C̲), 129.94 (Ph–C̲), 128.62 (Ph–C̲), 120.71 (Th–C̲5), 55.77 (Ph–C̲H_2_), 36.41 (C̲H(CH_3_)_2_), 26.99 (CH(C̲H_3_)_2_), and 14.40 (Th–C̲H_3_). ^11^B NMR (96 MHz, DMSO-*d*_6_) *δ* (ppm): −1.29 (singlet). ^19^F NMR (471 MHz, DMSO-*d*_6_) *δ* (ppm): −148.28 (singlet) (BF̲_4_). ESI-MS (+ve): 232.22 *m*/*z* [M − BF_4_^−^, C_14_H_18_NS]^+^; ESI-MS (−ve): 86.81 *m*/*z* [BF_4_]^−^.

#### 2.2.7 3-(4-(*tert*-Butyl)benzyl)-4-methylthiazol-3-ium tetrafluoroborate [^*t*^BBzTh][BF_4_] (3c)

Yield: 68%. ^1^H NMR (500 MHz, DMSO-*d*_6_) *δ* (ppm): 9.96 (s, 1H, Th–H̲), 7.73 (dd, *J* = 7.2, 1H, Ph–H̲), 7.71–7.62 (m, 3H, Ph–H̲), 7.61 (d, *J* = 7.3 Hz, 1H, Th–H̲), 5.39 (s, 2H, Ph–CH̲_2_), 3.77 (s, 3H, Th–CH̲_3_), 1.39 (s, 9H, C̲(CH̲_3_)_3_). ^13^C NMR (126 MHz, DMSO-*d*_6_) *δ* (ppm): 160.29 (Th–C̲2), 148.88 (Th–C̲4), 144.94 (Ph–C̲), 131.97 (Ph–C̲), 125.94 (Ph–C̲), 123.08 (Ph–C̲), 121.33 (Th–C̲5), 55.48 (Ph–C̲H_2_), 34.96 (C̲(CH_3_)_3_), 29.34 (C(C̲H_3_)_3_), and 14.53 (Th–C̲H_3_). ^11^B NMR (97 MHz, DMSO-*d*_6_) *δ* (ppm): −1.30 (singlet). ^19^F NMR (471 MHz, DMSO-*d*_6_) *δ* (ppm): −148.69 (singlet) (BF̲_4_). ESI-MS (+ve): 246.26 *m*/*z* [M − BF_4_^−^, C_15_H_20_NS]^+^; ESI-MS (−ve): 86.81 *m*/*z* [BF_4_]^−^.

#### 2.2.8 3-(4-Methylbenzyl)-4-methylthiazol-3-ium bis-((trifluoromethyl)sulfonyl)amide [MBzTh][Tf_2_N] (4a)

Yield: 79%. ^1^H NMR (300 MHz, DMSO-*d*_6_) *δ* (ppm): 10.01 (s, 1H, Th–H̲), 7.72 (d, *J* = 7.1 Hz, 1H, Ph–H̲), 7.65 (d, *J* = 7.2 Hz, 3H, Ph–H̲), 7.59 (d, *J* = 7.3 Hz, 1H, Th–H̲), 5.38 (s, 2H, Ph–CH̲_2_), 3.77 (s, 3H, Th–CH̲_3_), 2.64 (s, 3H, Ph–CH̲_3_). ^13^C NMR (75 MHz, DMSO-*d*_6_) *δ* (ppm): 158.30 (Th–C̲2), 149.97 (Tf_2_N–C̲F_3_), 144.92 (Th–C̲4), 137.59 (Ph–C̲), 130.70 (Ph–C̲), 128.82 (Ph–C̲), 126.32 (Ph–C̲), 117.49 (Th–C̲5), 56.44 (Ph–C̲H_2_), 22.47 (Ph–C̲H_3_), and 14.68 (Th–C̲H_3_). ^19^F NMR (565 MHz, DMSO-*d*_6_) *δ* (ppm): −81.66 (singlet) (Tf_2_N–CF̲_3_). ESI-MS (+ve): 204.09 *m*/*z* [M − Tf_2_N^−^, C_11_H_12_NS]^+^; ESI-MS (−ve): 279.93 *m*/*z* [C_2_F_6_NO_4_S_2_]^−^.

#### 2.2.9 3-(4-Isopropylbenzyl)-4-methylthiazol-3-ium bis-((trifluoromethyl)sulfonyl)amide [^i^PBzTh][Tf_2_N] (4b)

Yield: 74%. ^1^H NMR (200 MHz, CDCl_3_) *δ* (ppm): 10.06 (s, 1H, Th–H̲), 7.77 (d, *J* = 7.1 Hz, 1H, Ph–H̲), 7.70 (d, *J* = 7.1 Hz, 3H, Ph–H̲), 7.63 (d, *J* = 7.3 Hz, 1H, Th–H̲), 5.43 (s, 2H, Ph–CH̲_2_), 3.82 (s, 3H, Th–CH̲_3_), 3.37–3.29 (m, 1H, CH̲(CH_3_)_2_), 1.26 (d, *J* = 6.9 Hz, 6H, CH(CH̲_3_)_2_). ^13^C NMR (126 MHz, DMSO-*d*_6_) *δ* (ppm): 158.30 (Th–C̲2), 149.48 (Tf_2_N–C̲F_3_), 148.58 (Th–C̲4), 144.93 (Ph–C̲), 130.70 (Ph–C̲), 126.34 (Ph–C̲), 123.06 (Ph–C̲), 121.09 (Th–C̲5), 55.50 (Ph–C̲H_2_), 33.80 (C̲H(CH_3_)_2_), 22.47 (CH(C̲H_3_)_2_), and 14.41 (Th–C̲H_3_). ^19^F NMR (565 MHz, DMSO-*d*_6_) *δ* (ppm): −81.68 (singlet) (Tf_2_N–CF̲_3_). ESI-MS (+ve): 232.22 *m*/*z* [M − Tf_2_N^−^, C_15_H_20_NS]^+^; ESI-MS (−ve): 279.93 *m*/*z* [C_2_F_6_NO_4_S_2_]^−^.

#### 2.2.10 3-(4-(*tert*-Butyl)benzyl)-4-methylthiazol-3-ium bis((trifluoromethyl)sulfonyl)amide [^*t*^BBzTh][Tf_2_N] (4c)

Yield: 67%. ^1^H NMR (500 MHz, DMSO-*d*_6_) *δ* (ppm): 9.96 (s, 1H, Th–H̲), 7.73 (d, *J* = 7.2 Hz, 1H, Ph–H̲), 7.65 (dd, *J* = 7.3, 3H, Ph–H̲), 7.61 (d, *J* = 7.3 Hz, 1H, Th–H̲), 5.39 (s, 2H, Ph–CH̲_2_), 3.77 (s, 3H, Th–CH̲_3_), 1.39 (s, 9H, C̲(CH̲_3_)_3_). ^13^C NMR (126 MHz, DMSO-*d*_6_) *δ* (ppm): 160.28 (Th–C̲2), 149.43 (Tf_2_N–C̲F_3_), 148.76 (Th–C̲4), 144.94 (Ph–C̲), 131.97 (Ph–C̲), 125.95 (Ph–C̲), 123.08 (Ph–C̲), 121.08 (Th–C̲5), 55.58 (Ph–C̲H_2_), 34.96 (C̲(CH_3_)_3_), 29.34 (C(C̲H_3_)_3_), and 14.57 (Th–C̲H_3_). ^19^F NMR (565 MHz, DMSO-*d*_6_) *δ* (ppm): −81.34 (singlet) (Tf_2_N–CF̲_3_). ESI-MS (+ve): 246.24 *m*/*z* [M − Tf_2_N^−^, C_15_H_20_NS]^+^; ESI-MS (−ve): 279.93 *m*/*z* [C_2_F_6_NO_4_S_2_]^−^.

### 2.3. Biological studies

#### 2.3.1 Antimicrobial activity

The antimicrobial activity of newly synthesized benzyl thiazolium ionic liquids (BTILs) was investigated *in vitro* against the most common foodborne bacterial strains (*Staphylococcus aureus* (*S. aureus*, ATCC-25923) and *Escherichia coli* (*E. coli*, ATCC-25922)) in comparison with clinical drugs (Gentamycin (Gm) and Tetracyclin (TC)). These tests have been conducted in accordance with the standard antimicrobial assessment protocols we've described and used in our previous studies.^29^ The diameters of the zone of inhibition (ZOI, mm), values of minimum inhibitory concentration (MIC), and minimal bactericidal concentration (MBC) were used as markers for the antimicrobial efficacy of the tested compounds.

#### 2.3.2 *In vitro* antiproliferative performance

The antiproliferative activity of novel BTILs was tested *in vitro* against two cell lines: ovarian cancer (SKOV-3) and normal human skin fibroblast cells (HSF) using the standard MTT cytotoxicity assay described in our previous work.^30^ The cell viability ratios, half-maximal inhibitory concentration (IC_50_), and selectivity index (SI) were used as performance indices for the cytotoxicity of tested compounds. The clinical anticancer drug (cisplatin, CDDP) was used as a positive control.

## 3. Results and discussion

### 3.1. Design and synthesis of target benzyl thiazolium ionic liquids (BTILs)

**Scheme 1:**
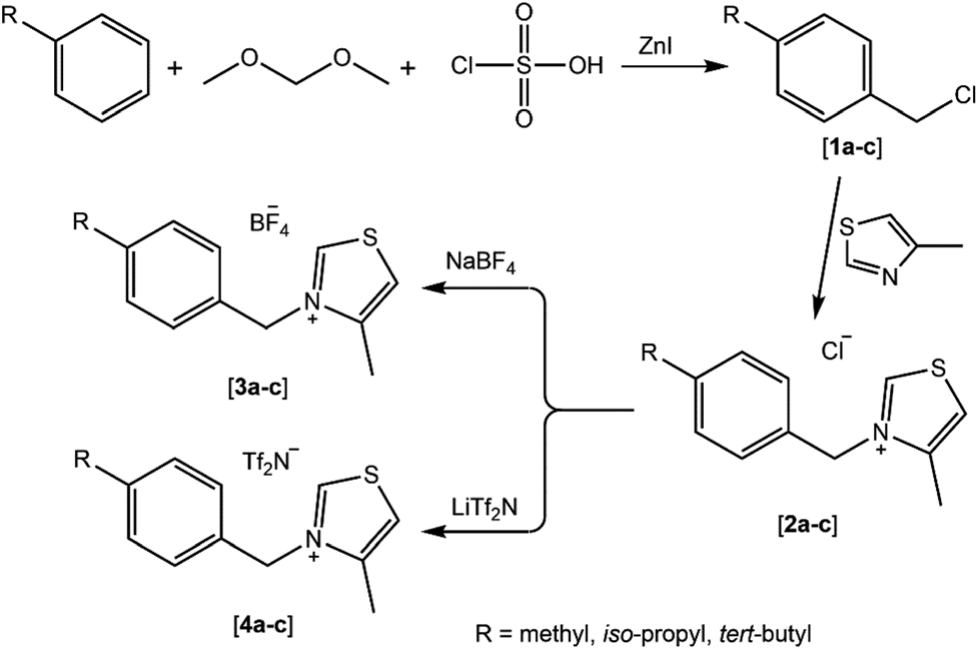
The stepwise pathway for the preparation of the targeted *N*-benzyl thiazolium ionic liquids.

As part of our continued interest in exploring and synthesizing novel heterocyclic derivatives for many uses, particularly in biological implementations,^31,32^ we present here a set of novel thiazolium ionic liquid derivatives for biological applications. The protocol adopted for the preparation of *N*-alkyl thiazolium ionic liquids from 1,3-thiazole involves three consecutive steps (see Scheme 1). In the first step, a chloromethylation reaction was performed on some alkylbenzenes (toluene, cumene, *tert*-butylbenzene) using a mixture of dimethoxymethane-chlorosulfonic acid-zinc iodide as a chloromethylating agent to yield the corresponding chlorobenzyl derivatives [1a–c]. Subsequently, the quaternization of 1,3-thiazole utilizing chlorobenzyl derivatives was conducted under reflux to produce the corresponding *N*-benzyl thiazolium chlorides [2a–c]. Eventually, these compounds were subjected to anion metathesis with tetrafluoroborate (BF_4_^−^) and bis-(trifluoromethanesulfonimide) (Tf_2_N^−^) at room temperature to yield the corresponding thiazolium ionic liquids [3a–c] and [4a–c] (Scheme 1).

All the synthesized thiazolium ionic liquids were obtained in good to excellent yields (58–79%) and their structural formulas were elucidated based on the spectral analyses (NMR (^1^H, ^13^C, ^11^B, ^19^F) and ESI-MS).

### 3.2. Physical properties of new thiazolium ionic liquids

#### 3.2.1 Physical state, solubility, and lipophilic properties

It is known that the pharmacokinetics and pharmacodynamics of any new pharmacological agent are tightly correlated to its aqueous solubility and lipophilic characteristics. Therefore, the aqueous solubility of the new thiazolium ionic liquids was investigated at ambient temperature. The solubility tests revealed that all the thiazolium ionic liquids are water-soluble, however, with a structure-dependent style. The extent of their aqueous solubility depends on the nature of both the cation and the anion (see Table 1). For instance, the benzyl thiazolium cation bearing methyl side chain and bound to a small hydrophilic anion (Cl^−^) (2a) was the most soluble one (log *S* = −3.383), whereas, the thiazolium cation that bearing the most hydrophobic side chain (*tert*-butyl) and bound to a hydrophobic anion (Tf_2_N^−^) (4c) was the most insoluble one (log *S* = −7.86).

**Table 1:** Physicochemical properties of new thiazolium ionic liquids^aa^

Compd	MW (g mol^−1^)	Nature	log *S*^b^	*C* log *P*^c^	*D* d (g cm^−3^)	*η* e (cP)	*T*_dec_ f (°C)
2a	239.76	Solid	−3.383	−1.182	NA	NA	NA
2b	267.82	Solid	−4.142	−0.254	NA	NA	NA
2c	281.84	Solid	−4.48	0.145	NA	NA	NA
3a	291.11	Oil	−4.569	−1.181	1.543	439.25 ± 5.3	340.3
3b	319.17	Semi-solid	−5.319	−0.250	NA	NA	NA
3c	333.20	Semi-solid	−5.653	0.147	NA	NA	NA
4a	484.45	Oil	−6.789	−0.352	1.536	428.75 ± 4.8	323.6
4b	512.50	Oil	−7.53	0.074	1.526	561.41 ± 6.1	298.7
4c	526.53	Oil	−7.86	0.205	1.531	587.35 ± 5.9	277.3

a MW, molecular weight.

b Calculated using ChemDraw 16.

c Calculated using ChemDraw 16.

d Measured density using COSMOtherm.

e Viscosity was measured at 50 °C and concentrations 0.5 mg mL^−1^ using a capillary viscometer.^34^

f Decomposition temperature taken from DTG curves.

As shown in Table 1, the *C* log *P* values revealed that the methylbenzyl-methylthiazolium cation is the least lipophilic cation with *C* log *P* values in the range of (−1.182)–(−0.352), depending on the type of counter anion. In contrast, replacing the methyl group with a *tert*-butyl group has remarkably increased the *C* log *P* values to be in the range of 0.145–0.205, depending on the bound anion, confirming their more lipophilic nature. Interestingly, the low *C* log *P* and high aqueous solubility values of thiazolium ionic liquids containing methylbenzyl-methylthiazolium and iso-propylbenzyl-methylthiazolium cations result in better bioavailability and reduced dosages required to obtain a good clinical response during treatment.^33^ Further, increasing the lipophilicity of the cation results in strong interactions between the ionic liquids and the outer lipophilic layer of the microbial cell surface.^19^

#### 3.2.2 Viscosity and thermal stability

Because drug viscosity has a strong influence on its pharmacokinetics, molecular diffusion, and biological response, the viscosities of new thiazolium ionic liquids were measured at 25 °C. As shown in Table 1, all thiazolium derivatives exhibited moderately high viscosity values (428.75–587.35 cP). Also, the viscosity of new benzyl thiazolium ionic liquids depends on the intrinsic structural features of the cation and anion. For instance, the ionic liquid 4a that composed of the simplest cation (methylbenzyl-methylthiazolium) and bis-(trifluoromethanesulfonimide) anion exhibited the lowest viscosity (428.75 cP) amongst all tested ionic liquids. Conversely, IL 4c having a *tert*-butylbenzyl group exhibited the highest viscosity (587.35 cP). This behavior can be explained based on the fact that the extremely hydrophobic Tf_2_N^−^ anion, enriched with fluorine electrons, exerts higher ion–ion interactions with hydrophobic *tert*-butylbenzyl-methylthiazolium cation, resulting in an increase in viscosity.^35^

**Fig. 1:**
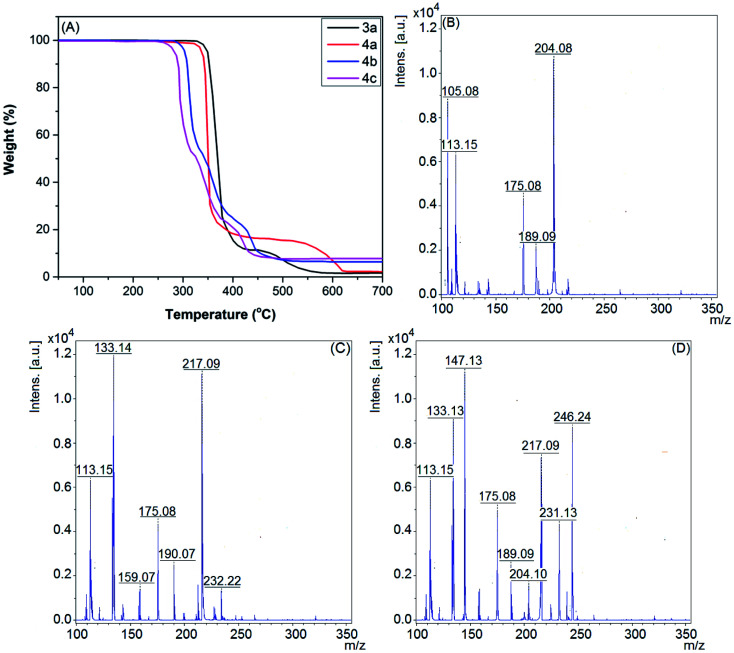
(A) TG thermograms for the new room-temperature thiazolium ionic liquids; (B–D) electrospray ionisation mass spectra of RTTILs 4a–c in a positive mode (ESI-MS (+ve)).

The thermogravimetric (TG) curves of the room temperature thiazolium ionic liquids (RTTILs) (3a, 4a–c) (Fig. 1A) were employed for the investigation of their thermal stabilities. It is evident that they are thermally stable up to 270 °C and then undergo a sharp degradation phase in the temperature range of 270–350 °C. As shown in Fig. 1A, RTTILs with long side chains linked to the N-atom of the thiazolium ring (4b,c) exhibited more complicated thermal decomposition styles and lower initial decomposition temperatures as compared to those with methyl groups (3a and 4a). For instance, the TGA diagram of compound 4a shows two distinct thermal degradation phases. The first main decomposition phase (∼81% weight loss) starts at 323 °C, followed by a modest decomposition stage between 496 and 618 °C. Conversely, IL 4b exhibits three characteristic decomposition steps. The first thermal degradation step begins at 298 °C (∼52% weight loss) and is followed by two smaller degradation steps at 353 and 418 °C, respectively.

### 3.3. Structural characterization

#### 3.3.1 Mass spectrometery

The electrospray ionization mass spectra (ESI-MS) of the newly synthesized TILs can offer an informative tool to get an initial perception of the structural characteristics of their cations and anions. Since most of the new TILs contain the same cations or anions, they exhibit identical ESI-MS. Therefore, the ESI-MS of TILs 4a–c were studied as a represented series for new TILs (see Fig. 1B–D). As shown in their spectra, the main distinctive peaks were observed at *m*/*z* values of 204.09, 232.22, and 246.24, corresponding to the molar mass of a single-charged cation which formed owing to the removal of the bound anion, [M − Tf_2_N^−^]^+^.

In addition, common fragmentation peaks were observed in the mass spectra of the thiazolium ionic liquids as a result of the consecutive liberation of alkyl side chains, methylthiazole and/or methylthiazolium fragments from the parent molecule, [M − Tf_2_N^−^ − R˙]^+^, [M − Tf_2_N^−^ − MeTh]^+^, and [M − Tf_2_N^−^ − MeThH˙]^+^.

#### 3.3.2 FTIR spectroscopy

**Fig. 2:**
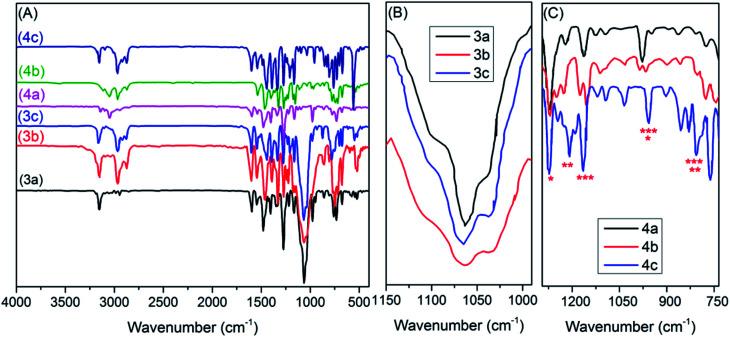
FTIR spectra of the new thiazolium ionic liquids, showing the vibrational bands distinctive for structural characteristics of the cations (A); BF_4_^−^ anion (B); and Tf_2_N^−^ anion (C).

In an effort to understand some of the structural characteristics of the cations and anions of the new thiazolium ionic liquids (TILs), their FTIR spectra were acquired (Fig. S1–S5, ESI†) and (Fig. 2). The thiazolium ILs' spectra show absorption bands similar to those distinctive to imidazolium-based ILs.^36,37^ Initially, it can be seen in the FTIR spectra of TILs (Fig. 2A) that the absorption bands distinctive for the benzylthiazolium cation are centered around 3100, 2950, 1590, 1240, 855, and 700 cm^−1^ which could be assignable to the different vibrational modes of C2–H of the thiazole ring,^33^ C–H of an alkyl group, C=N/C–S of thiazolium cation,^38^ and benzyl moiety,^33^ respectively. On the other hand, the characteristic bands for anions of TILs 3a–c are readily observable in a 1062 ± 2 cm^−1^ region (see Fig. 2B) that can be assigned to the vibrational modes of [BF_4_^−^], as the thiazolium cations do not exhibit strong absorption bands in this region.^39^ Conversely, the inspection of FTIR spectra for TILs 4a–c (Fig. 2C) revealed remarkable changes in the characteristic bands for anion (TN_2_f) such as the emergence of several peaks in the regions: 1272 ± 2 cm^−1^ assigned to *ν*_as_(CF_3_) + *ν*_as_(SO_2_); 1223 ± 3 cm^−1^ due to *ν*_s_(CF_3_) + *ν*_s_(SO_2_); 1155 ± 5 cm^−1^ characteristic for *ν*_as_(CF_3_) + *ν*(C–S); 1017 ± 2; 905 ± 5 cm^−1^ distinctive for *ν*(N–S); 796 ± 3 cm^−1^ due to *δ*(CF_3_); and 747 ± 3 cm^−1^ assigned to *ν*(C–S), of the TN_2_f anion.^40,41^ In general, the new thiazolium ILs exhibited FTIR signatures comparable to those previously reported in the literature.^33^

#### 3.3.3 NMR spectroscopy

**Fig. 3:**
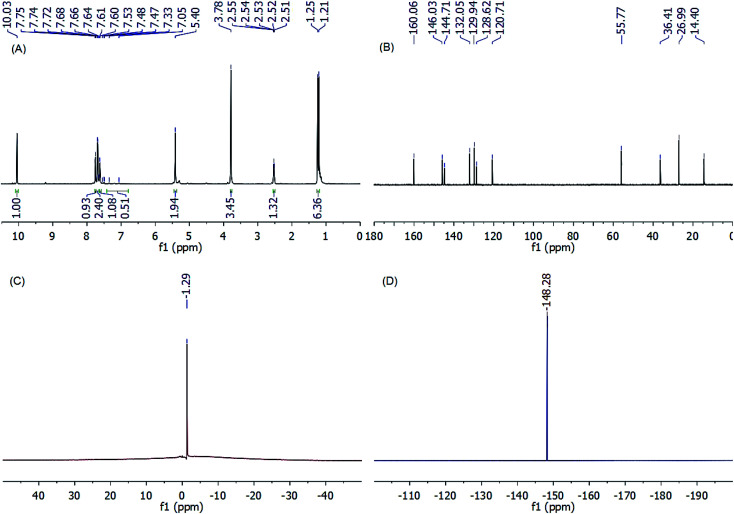
(A) ^1^H NMR (200 MHz, CDCl_3_), (B) ^13^C NMR (125 MHz, CDCl_3_), (C) ^11^B NMR (96 MHz, DMSO-*d*_6_), and (D) ^19^F NMR (471 MHz, DMSO-*d*_6_) of TIL (3b).

The NMR spectra of the new thiazolium ionic liquids (Fig. S7–S28, ESI†) were used to confirm their successful formation and give an emphatic imagine of the structure of their cations and anions. For instance, the ^1^H NMR spectrum of TIL 3b (Fig. 3A) shows a singlet at 10.03 ppm assigned to the resonance of thiazolic proton (S–C–H). In addition, a set of signals was observed in the chemical shift region of 7.75–7.33 ppm, corresponding to the resonances of protons linked to the thiazolium and phenyl rings. Also, the benzylic and methyl protons emerged as two singlet peaks at 5.40 and 3.78 ppm, respectively. Further, it can observe a set of doublet (1.23 ppm, 6H) along with a methine septet (3.33 ppm) corresponding to the isopropyl group.

On the other hand, the emergence of eleven distinct carbon peaks in the ^13^C NMR spectrum of TIL 3b validates the molecular structure of the synthesized ionic liquid. As shown in Fig. 3B, the carbon signal at 14.4 ppm was assigned to the CH_3_ group of methylthiazole moiety, whereas, the three peaks at 26.99, 55.77, and 36.41 ppm were ascribed to the resonance of methyl and methine carbons of the isopropyl group and benzylic carbon atom, respectively. The emergence of four characteristic carbon signals at 144.71, 132.05, 129.94, and 128.62 confirms the presence of a benzyl ring on the thiazole ring. Another three peaks were observed in the low-field region at *δ* 120.71, 160.06, and 146.03 ppm corresponding to N–C̲–H, N–C̲–Me, and S–C̲–H of thiazolium ring, respectively.

Notable, the structural identities of the counter-anions for the synthesized thiazolium ionic liquids were identified using negative-mode electrospray mass spectrometry (ESI-MS (−ve)), ^11^B NMR, and ^19^F NMR analyses. Where, the ESI-MS (−ve) of the TILs showed major common peaks at *m*/*z* 34.97 for Cl^−^, 86.81 for BF_4_^−^, and 279.93 for Tf_2_N^−^. Additionally, the emergence of a singlet (*δ* −1.29 ppm) in ^11^B NMR spectra, as well as a singlet (*δ* −148.28 ppm) in the ^19^F NMR spectra of TILs 3a–c (ESI†), confirms the presence of BF_4_^−^ as a counter anion for these ILs. Conversely, the emergence of a singlet at *δ* −81.66 ppm in the ^19^F NMR spectra of TILs 4a–c (ESI†) is indicative of the binding of these ILs to Tf_2_N^−^ as a counter anion.

### 3.4. Antimicrobial study

The amazing antimicrobial characteristics of ILs^42^ have unlocked new vistas in tackling the current challenges related to fighting antibiotic-resistant pathogens. Motivated by these facts, the antibacterial activities of the newly synthesized thiazolium ionic liquids were investigated against the most common foodborne bacteria, *S. aureus* and *E. coli*.

#### 3.4.1 Well diffusion assay

**Fig. 4:**
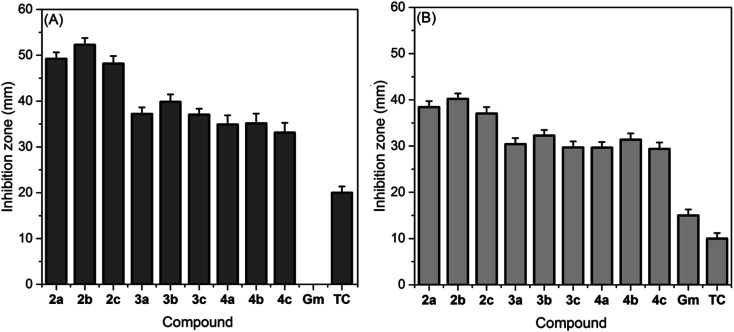
Zone of inhibition (ZOI, mm) graph for the newly synthesized thiazolium ionic liquids against (A) *S. aureus* (ATCC-25923) and (B) *E. coli* (ATCC-25922).

The antibacterial activities of novel TILs against *S. aureus* and *E. coli* were screened *in vitro*, as compared to clinical antibiotics (Gentamycin (Gm) and Tetracyclin (TC)). The zone of inhibition (ZOI) values (see Fig. 4) demonstrate that all TILs can significantly inhibit the proliferation of bacterial cells with bacterium strain-dependent and structure-dependent performances. In general, the Gram-negative bacteria (*E. coli*) (see Fig. 4B) exhibited more tolerance toward all treatments than the Gram-positive ones (*S. aureus*) (see Fig. 4A). This could be attributed to the bacterial cell membrane's interaction with TILs. Gram-negative bacteria' outer membranes, which are composed of impermeable lipopolysaccharides (LPS), phospholipids, and lipoproteins, may act as a barrier to the entry of antimicrobial molecules, whereas Gram-positive bacteria's outer membranes have no LPS layer and are composed only of a thick peptidoglycan layer which can easily absorb and transfer such antimicrobials to the inner cell membrane.^43^ A comparison of antibacterial activities for any members of the same series demonstrated that the slight increase in the alkyl chain length has little effect on the antibacterial efficacy, whereas, the change of counter anion significantly affects the antibacterial activity for TIL. For example, the ZOI diameters for [MBzTh][BF_4_], [^i^PBzTh][BF_4_], and [^*t*^BBzTh][BF_4_] against *S. aureus* were 39.21, 41.86, and 39.02 mm, respectively. Conversely, the ZOI values for [^i^PBzTh][Cl], [^i^PBzTh][BF_4_], and [^i^PBzTh][Tf_2_N] against *S. aureus* were 32.13, 41.86, and 52.29 mm, respectively. These findings are fully consistent with literature that documented that the antimicrobial activity of ILs linked to short alkyl chains is found to depend on the chaotropicity of the anion.^44^ Notably, the anion chaotropicity refers to its ability to destroy the hydrogen bonding network between water molecules or in a macromolecular structure. As a result, it is strongly correlated to the number of active H-bonding sites in the anion.^45^ In this scenario, the toxicity of TILs of the same cation toward the bacterial species was as follows; [Tf_2_N]^−^ ≫ [BF_4_]^−^ ≥ [Cl]^−^. This matches the Hofmeister series and agrees with earlier studies related to the chaotropicity and kosmotropicity of anions of ILs. [^i^PBzTh][Tf_2_N] was the most active bactericidal agent in all cases, with maximum ZOIs > 40 mm in all cases.^45^

#### 3.4.2 Minimal inhibitory/bactericidal concentrations (MIC/MBC)

As evident in Table 2, the results of MIC and MBC are in general accordance with the Well diffusion assay. Again, *S. aureus* is more sensitive toward treatments with TILs (MIC/MCB = 0.75/0.75–4.05/4.25 mM) than *E. coli* (MIC/MCB = 2.55/3.25–6.75/12.25 mM). Amongst new thiazolium ionic liquids, [^i^PBzTh][Tf_2_N] exhibited the most potent bactericidal activity and showed the least MIC/MBC values toward both *S. aureus* (0.75/0.75 mM) and *E. coli* (2.55/3.25 mM). On the other hand, [MBzTh][Cl] is the weakest antibacterial agent with MIC/MBC values of (4.05/4.25 mM) and (6.75/12.25 mM) against *S. aureus* and *E. coli*, respectively. Overall, the small lengthening of the alkyl side chain on the thiazolium cation showed little effect on MIC and MBC values, whereas, the anion metathesis had a great impact. MIC and MBC results revealed that the Tf_2_N-based TILs are the strongest antibacterial agents against tested pathogens. In the literature, quantitative information on the antimicrobial activity of short-chained and benzyl-functionalized ionic liquids is scarce. In this context, few studies have reported the antibacterial impact of 1-alkyl-3-methylimidazolium ionic liquids, bound to different anions, upon various pathogenic bacteria. The findings of these studies revealed that the 1-hexyl-3-methylimidazolium bis((trifluoromethyl)sulfonyl)amide is the most active one with MIC/MBC_*E.coli*_ 24.59/24.59 mM and MIC/MBC_*S.aureus*_ 9.13/9.13 mM,^46,47^ which are significantly higher than the values obtained for the new TILs.

**Table 2:** MIC and MBC values (mM) for the newly synthesized thiazolium ionic liquids against *S. aureus* (ATCC-25923) and *E. coli* (ATCC-25922), in comparison to clinical antibiotics^a^

TIL	*S. aureus*		*E. coli*	
	MIC ± SD	MBC ± SD	MIC ± SD	MBC ± SD
2a	4.05 ± 0.29	4.25 ± 0.31	6.75 ± 0.35	12.25 ± 0.36
2b	2.35 ± 0.27	2.35 ± 0.28	5.25 ± 0.31	5.25 ± 0.29
2c	3.75 ± 0.19	3.75 ± 0.14	6.25 ± 0.27	6.25 ± 0.29
3a	3.25 ± 0.19	3.25 ± 0.21	5.75 ± 0.41	5.75 ± 0.45
3b	2.56 ± 0.13	2.56 ± 0.15	5.25 ± 0.29	6.25 ± 0.23
3c	3.12 ± 0.19	3.12 ± 0.14	6.50 ± 0.39	6.50 ± 0.39
4a	1.05 ± 0.19	1.05 ± 0.15	3.37 ± 0.31	6.25 ± 0.29
4b	0.75 ± 0.11	0.75 ± 0.09	2.55 ± 0.42	3.25 ± 0.51
4c	1.25 ± 0.30	1.25 ± 0.33	3.50 ± 0.49	3.46 ± 0.47
Gentamycin	NA	NA	6.85 ± 0.63	NA
Tetracycline	7.20 ± 0.66	NA	NA	NA

a NA = not assigned.

### 3.5. Cytotoxicity study

#### 3.5.1 Antiproliferative activity

**Fig. 5:**
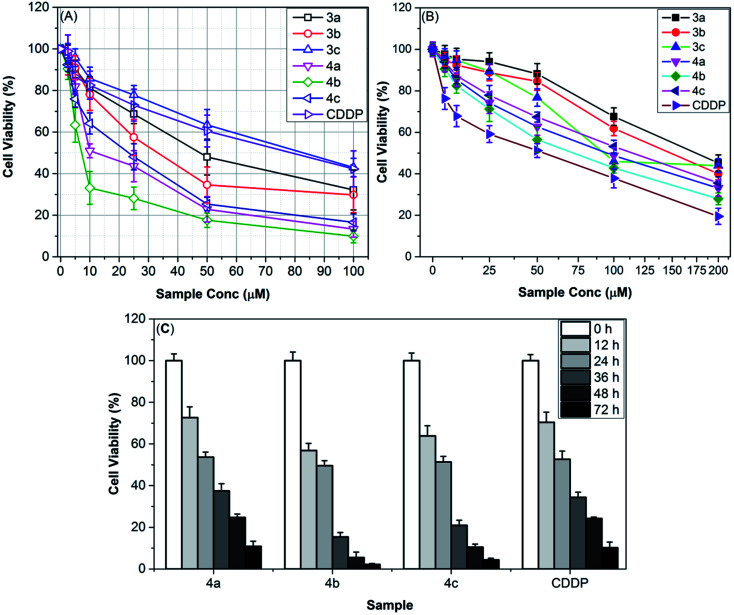
The effect of different doses (0–200 μM) of TILs (3a–c) and (4a–c) on the viability of (A) ovarian carcinoma cell lines (SKOV-3) and (B) human skin fibroblast (HSF) cells after 24 h of treatment as compared to the clinical antitumor drug (cisplatin, CDDP). (C) Time-dependent antiproliferative performance of thiazolium ILs (4a–c) against SKOV-3 cell lines at their respective IC_50_ dosages (4a, 19.31 μM; 4b, 7.37; 4c, 15.22 μM) or CDDP (79.48 μM) during 72 h of treatment.

The use of ionic liquids (ILs) in pharmaceutical and medicinal chemistry has recently piqued the interest of researchers across the globe.^12^ In this scenario, several studies have documented the anticancer potential of ILs.^20,48^ In order to find potential safe anticancer candidates, the cytotoxicity of imidazolium, ammonium, pyridinium, and phosphonium ILs was tested against over 60 human cancer cell lines.^12,49^ The findings of these studies revealed the promising anticancer potential of ILs against several tumor cell lines. Besides, the cytotoxicity of ILs is strongly correlated to the counter anion. For instance, the cytotoxicity of 1-alkyl-3-methylimidazolium cation [RMIM]^+^ follows the following trend; [RMIM][Tf_2_N] ≫ [RMIM][BF_4_] > [RMIM][Cl].^50^ To our knowledge, no previous studies have been reported so far for using thiazolium ILs for anticancer purposes. These facts have inspired us to design and study the antiproliferative activity of new thiazolium ILs tethering different anions (BF_4_ (3a–c) and Tf_2_N (4a–c)) against ovarian carcinoma cell lines (SKOV-3) and normal human skin fibroblast cells (HSF). The 3-(4,5-dimethylthiazol-2-yl)-2,5-diphenyltetrazolium bromide (MTT) assay was used to carry out this study, with cisplatin (CDDP) serving as a positive control. Initially, the impacts of serial concentrations (2.5–100 μM) of the thiazolium ILs on the viability of the SKOV-3 cell lines were assessed as compared to CDDP. The findings of this evaluation (Fig. 5A) demonstrated that the SKOV-3 cellular viability has remarkably diminished in the presence of increasing concentrations of TILs or CDDP, with a dose-dependent performance, reaching a minimum viability value with a maximum dose (100 μM). In general, TILs bound to Tf_2_N anion (4a–c) inhibited the proliferation of SKOV-3 cells more effectively than the corresponding tetrafluoroborate analogues (3a–c). For instance, [^i^PBzTh][Tf_2_N] (4b) is the most cytotoxic one and can reduce the SKOV-3 cellular viability to a minimum value (10.5 ± 1.7%, Fig. 5A) at its treatment dosage of 100 μM. Conversely, [MBzTh][BF_4_] (3a) is the least cytotoxic one as it induces only a 54.6% reduction in SKOV-3 viability using the same dosage (Fig. 5A).

#### 3.5.2 Selectivity of new TILs toward cancer cells over normal ones

To explore the selectivity of the newly synthesized TILs for attacking cancer cells over normal ones, the effects of serial concentrations (0–200 μM) of these TILs on the proliferation of normal human skin fibroblast (HSF) cells were also studied for a period of 24 h. It can be seen in Fig. 5B that all TILs exhibited little dose-dependent effects on the viability of normal cells (HSF), in comparison to their impacts on cancer cells (SKOV-3) under the same conditions. For example, treatment of both cell lines with 100 μM of [^i^PBzTh][Tf_2_N] (4b) under the same conditions resulted in about 90% and 58% reduction of SKOV-3 and HSF cellular viability, respectively. [MBzTh][BF_4_] (3a) showed the lowest antiproliferative activity against HSF cells, inducing minimum HSF viability (45.5 ± 4.1%, Fig. 5B) at a dose of 200 μM. Interestingly, all tested TILs are less toxic to normal cells than CDDP.

As shown in Table 3, the IC_50_ values of the new TILs against both cell lines are in accordance with the aforementioned hypotheses. It can be seen that the IC_50_ values for these TILs against ovarian cancer cells were in the range of 7.37–77.95 μM (IC_50_ for CDDP 79.56 μM), whereas, the IC_50_ values against HSF cells were 75.42–191.26 μM (IC_50_ for CDDP 58.28 μM), indicating that SKOV-3 cells are more sensitive than HSF toward treatments with tested thiazolium ILs. Besides, again, Tf_2_N-tethered thiazolium ILs (4a–c) are more toxic for SKOV-3 cells (IC_50_ 7.37–21.31 μM) as compared to the corresponding tetrafluoroborate salts (3a–c) (IC_50_ 35.73–77.95 μM).

**Table 3:** Values of IC_50_ (μM) and selectivity index (SI) for the synthesized thiazolium compounds against ovarian cancer (SKOV-3) and normal human skin fibroblast (HSF) cell lines, in comparison with a clinical drug (CDDP)

Cell line	IC_50_ (μM) ± SD						
	3a	3b	3c	4a	4b	4c	CDDP
SKOV-3	77.95 ± 1.35	35.73 ± 1.03	51.07 ± 2.33	21.31 ± 1.05	7.37 ± 1.11	15.22 ± 1.23	79.48 ± 1.18
HSF	177.52 ± 8.79	152.24 ± 9.15	191.26 ± 9.85	113.19 ± 7.39	75.42 ± 8.09	91.81 ± 7.35	58.28 ± 6.67
SI	2.28	4.26	3.76	5.87	10.23	6.03	0.73

Furthermore, the selectivity levels of the tested thiazolium ILs for the cancer cells (SKOV-3) over normal ones (HSF) can be investigated by calculating the selectivity index (SI) for the tested TILs using the following equation;^51^
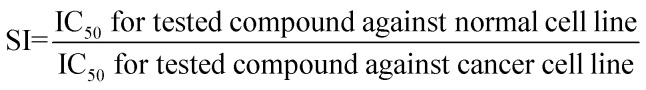


It is evident in Table 3 that [^i^PBzTh][Tf_2_N] (4b) (SI = 10.23) exhibited the highest level of selectivity for attacking SKOV-3 cells over normal cells (HSF). Thus, [^i^PBzTh][Tf_2_N] could act as a promising anti-ovarian cancer agent without inducing harm to healthy human skin fibroblasts. Consequently, this study offers the first ionic liquid that can serve as an anti-ovarian cancer agent which may open a wide window for exploring a new generation of TILs-based anti-ovarian cancer agents.

To study the effect of the duration of remediation using TILs on their anti-ovarian cancer activity, the SKOV-3 cell lines were injected with aliquot IC_50_ doses of the most potent thiazolium ILs (4a–c) and cisplatin and incubated for 72 h. At definite time intervals, the SKOV-3 cell viability has been measured. As shown in Fig. 5C, the viability of SKOV-3 cells was dramatically reduced after incubation for 24 h with the most potent anti-ovarian cancer candidate [^i^PBzTh][Tf_2_N] (4b) and virtually totally suppressed after 72 h, reaching a minimum viability (2.2 ± 0.43%). CDDP also caused a time-dependent decrease in SKOV-3 cell viability, albeit with less efficiency than IL 4b, which caused limited SKOV-3 survival at 72 h (10.2 ± 2.7%).

#### 3.5.3 Proposed mechanism for anticancer activity of new thiazolium compounds

**Fig. 6:**
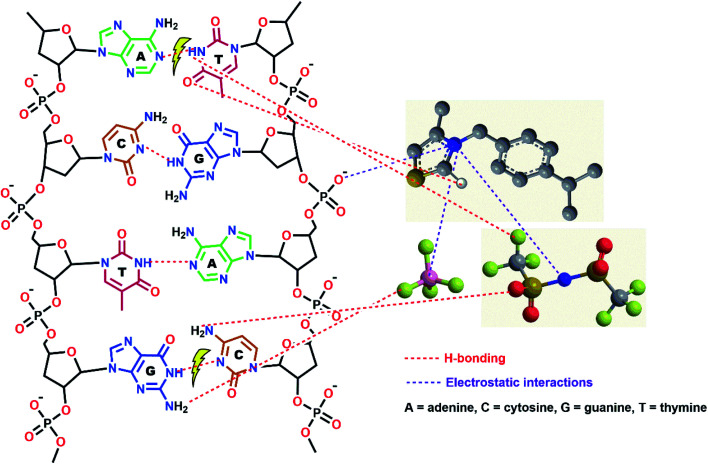
The proposed mechanism for anticancer activity of new thiazolium ILs shows: (i) hydrogen-bonding between the active H-bonding sites (HBSs) of the TILs backbone (C2–H, and F) and the HBSs sites of DNA nucleobases (NH, NH_2_, N, and C=O); (ii) electrostatic attraction between the cationic thiazolium ring and the phosphate groups of DNA. These different types of interaction may result in a malfunction of DNA or fission of its double-strand.

The amphiphilic nature of the new thiazolium derivatives enables them to strongly interact with DNA and phospholipid bilayers of the cell membrane through electrostatic interactions between the cationic thiazolium ring and phosphate groups scattered on the surface of this pharmacological target (DNA), causing their malfunctions.^19^ In addition, thiazolium, which is similar to imidazolium ILs, may also cause oxidative stress, mitochondrial dysfunction, and death in cancer cells.^19^ The anion-dependent cytotoxicity of new thiazolium ionic liquids could be ascribed to various effects such as their lipophilicity, hydration state, hydrolytic cleavage vulnerability, and hydrogen bonding capability. The over-additive anticancer effect of Tf_2_N-based thiazolium ILs (4a–c) could be attributed to the temporary production of stable ion pairs in aqueous solutions, which can result in much greater bioavailability of the particular ionic liquid, enhancing membrane interactions and causing harsh cytotoxic effects.^46^ Further, the Tf_2_N anion possesses a number of active H-bonding sites (HBSs) (6 F, 2 O, N) more than that of BF_4_ which contains only 4 fluorine atoms as HBSs. Consequently, Tf_2_N-linked TILs (4a–c) can form more H-bonds with nucleobases of DNA than those formed by BF_4_ analogies (3a–c) (See Fig. 6). As [Tf_2_N]^−^ has the highest tendency to form H-bonds with pharmacological targets and exhibits high hydrophobicity as well, it can disrupt the H-bonding network in biological macromolecules (DNA and proteins) by its higher chaotropic activity.^47^

## 4. Conclusion

A novel series of thiazolium ionic liquids (TILs) bound to chloride (2a–c), tetrafluoroborate (BF_4_) (3a–c), and bis-(trifluoromethanesulfonimide) (Tf_2_N) anions (4a–c) has been successfully synthesized. The new TILs were obtained in good yields and their structural formulas were investigated based on microanalytical, spectral (FTIR, UV-Vis, NMR (^1^H, ^13^C, ^19^F, ^11^B) and ESI-MS), and thermal analyses. The *in vitro* antibacterial study revealed that the respective chaotropic activity of the IL anion significantly influences their respective antibacterial performance. According to the Hofmeister series and its related impacts, ILs with identical cations become more bactericidal upon binding with a strong chaotropic anion (eg, TN_2_f). The *in vitro* toxicity of the newly synthesized TILs toward the ovarian carcinoma cell line (SKOV-3) was assessed using the MTT assay, in comparison to cisplatin (CDDP). The selectivity of new TILs for attacking cancer cells over healthy ones (*e.g.* human skin fibroblast cells (HSF)) was also investigated. The findings revealed that all the TILs have the capacity to induce a dose- and time-dependent decline in the cell viability of SKOV-3, with a preferable efficacy for Tf_2_N-linked TILs (4a–c). In addition, the new compounds showed excellent selectivity for cancer cells (SKOV-3) over healthy cells (HSF), as revealed by their selectivity index values. [^i^PBzTh][Tf_2_N] (4b) is the most cytotoxic one and can reduce the SKOV-3 cellular viability to a minimum value (10.5 ± 1.7%) at 100 μM dose. Besides, it exhibited the highest level of selectivity (SI = 10.23) for attacking SKOV-3 cells over normal cells (HSF). Therefore, our findings demonstrated the potential application of [^i^PBzTh][Tf_2_N] as a novel anti-ovarian cancer agent, in the future. However, further work on remaining issues for these thiazolium ILs is still needed, which will be the aim of our future studies.

## Conflicts of interest

We wish to confirm that there are no known conflicts of interest associated with this publication and there has been no significant financial support for this work that could have influenced its outcome.

## Supplementary Material

RA-012-D1RA07128A-s001
