# Supplementary material for: Fine-tuning of the pharmacological potential of novel thiazolium ionic liquids by anion alteration
Source: RSC Adv. 2021 Dec 22;12(1):458–69. doi: 10.1039/d1ra07128a (PMC8978639; doi:10.1039/d1ra07128a)
Supplement: RA-012-D1RA07128A-s001 [file RA-012-D1RA07128A-s001.pdf]

Electronic Supplementary Material (ESI) for RSC Advances.
This journal is © The Royal Society of Chemistry 2021

Fine-tuning of pharmacological potentials of novel thiazolium ionic liquids by anion alteration

Mohammad Y. Alfaifi,[a] Ali A. Shati, [a] Serag Eldin I. Elbehairi,[b] Reda F. M. Elshaarawy,[c,d,]* Emad M. Gad[e]

[a] Biology Department, Faculty of Science, King Khalid University, 9004 Abha, Saudi Arabia.
[b] Cell Culture Lab, Egyptian Organization for Biological Products and Vaccines (VACSERA Holding Company), Giza 12311, Egypt.
[c] Chemistry Department, Faculty of Science, Suez University, 43533 Suez, Egypt.
[d] Institut für Anorganische Chemie und Strukturchemie, Heinrich-Heine Universität Düsseldorf, Düsseldorf, Germany.
[e] Chemistry Department, Faculty of Science, Suez Canal University, Ismalia, Egypt.

Corresponding author:
RFME; reda.elshaarawy@suezuniv.edu.eg; reel-001@hhu.de

Contents:

1- **Materials and instrumentation**
2- **Synthesis of 1-alkyl-4-(chloromethyl)benzene (1a-c)**
3- **Figures**

1. Materials and instrumentation

Chemicals were obtained from the following suppliers and used without further purification: toluene, cumene, tert-butylbenzene, 4-methylthiazole, sodium tetrafluoroborate, lithium bis(trifluoromethanesulfonimide), and anhydrous $MgCl_2$ (Sigma–Aldrich); Zinc iodide (Acros).

Melting points were measured using a BÜCHI Melting point B-540 apparatus; all melting points were measured in open glass capillaries and are uncorrected. Elemental analyses for C, H, N, were performed with a Perkin–Elmer 263 elemental analyzer. FT-IR spectra were recorded on a BRUKER Tensor-37 FT-IR spectrophotometer in the range 400–4000 cm^{-1} as KBr disc in the 4000-550 cm^{-1} region with 2 cm^{-1} resolution or with an ATR (attenuated total reflection) unit (Platinum ATR-QL, diamond). For signal intensities the following abbreviations were used: br (broad), sh (sharp), w (weak), m (medium), s (strong), vs (very strong). UV/Vis spectra were measured at 25 °C in ethanol (10^{-5} mol/L) on a Shimadzu UV-2450 spectrophotometer using quartz cuvettes (1 cm). NMR-spectra were obtained with a Bruker Avance DRX200 (200 MHz for ^{1}H) or Bruker Avance DRX500 (125, 97 and 470 MHz for ^{13}C, ^{11}B and ^{19}F respectively) spectrometer with calibration to the residual proton solvent signal in DMSO-d_6 (^{1}H NMR: 2.52 ppm, ^{13}C NMR: 39.5 ppm), $CDCl_3$ (^{1}H NMR: 7.26 ppm, ^{13}C NMR: 77.16 ppm) against TMS ($\delta = 0.00$ ppm) for ^{1}H and ^{13}C, 85% phosphoric acid ($\delta = 0.00$ ppm) for ^{31}P and $CFCl_3$ ($\delta = 0.00$ ppm) for ^{19}F NMR. Multiplicities of the signals were specified s (singlet), d (doublet), t (triplet), q (quartet) or m (multiplet). The ESI-MS of the synthesized compounds were acquired in the linear mode for positive ions on a UHR-QTOF maXis 4G (Bruker Daltonics) and BRUKER Ultraflex MALDI-TOF instrument equipped with a 337 nm nitrogen laser pulsing at a repetition rate of 10 Hz. The 2+ charge assignment of ions in HR-ESI-MS was confirmed by the m/z = 0.5 difference between the isotope peaks (x, x+1, x+2). Peaks with chlorine showed the isotope ratio $^{35/37}Cl$ = 75.8:24.2.

2. Synthesis of 1-alkyl-4-(chloromethyl)benzene (1a-c)

General procedure: A flask was charged with 5 mol% of ZnI_2 (1.3 mmol), chlorosulfonicacid (31 mmol) and CH_2Cl_2 (30 mL), followed by dropwise addition of dimethoxymethane (31 mmol) at -10 °C. After stirring the reaction mixture at -10°C for 30min, the aromatic compound (26 mmol) was slowly added.The resulting mixture was then stirred at 5-10 °C for the time 0.5 - 2 h. The reaction was monitored by TLC analysis. After

completion, the reaction was quenchedby addition of water (10 mL) in an ice bath. After extractionwith CH_2Cl_2 (3 x 20 mL), the organic phase was washedwith 5% sodium carbonate solution (2 x 10 mL), water (2 x10 mL) and brine (2 x 20 mL), then evaporated to drynessunder reduced pressure. The residue was purified by flashcolumn chromatography on a silica gel using petroleumether (boiling range: 60-90°C) and ethyl acetate as eluents to give the desired product.

1-(Chloromethyl)-4-ethylbenzene (**1a**) [24]: Colorless liquid. ^{1}H NMR (500 MHz, $CDCl_3$) δ (ppm): δH 7.41 (d, *J* = 8.1 Hz, 2H), 7.30 (d, *J* = 8.1 Hz, 2H), 4.67 (s, 2H), 2.68 (s, 3H); 13C NMR (125 MHz, $CDCl_3$) δ (ppm): 141.8, 131.5, 128.8, 127.9, 46.2, 28.4.

1-(Chloromethyl)-4-isopropylbenzene (**1b**) [24]: Colorless liquid. ^{1}H NMR (500 MHz, $CDCl_3$) δ (ppm): 7.41 (d, *J* = 8.1 Hz, 2H), 7.32 (d, *J* = 8.1Hz, 2H), 4.66 (s, 2H), 3.05–2.93 (m, 1H), 1.35 (d, *J* = 7.4 Hz, 6H); 13C NMR (125 MHz, $CDCl_3$) δ (ppm): 146.3, 138.7, 128.6, 126.7, 46.0, 33.9, 23.9.

*1-(*tert*-Butyl)-4-(chloromethyl)benzene* (**1c**) [24]: Colorless liquid. ^{1}H NMR (500 MHz, $CDCl_3$) δ (ppm): δH 7.46 (d, *J* = 8.4 Hz, 2H), 7.42–7.39 (m, 2H), 4.64 (s, 2H), 1.40 (s, 9H); 13C NMR (125 MHz, CDCl3) δ (ppm):151.4, 134.5, 128.3, 125.6, 46.0, 34.5, 31.3.

3. Figures

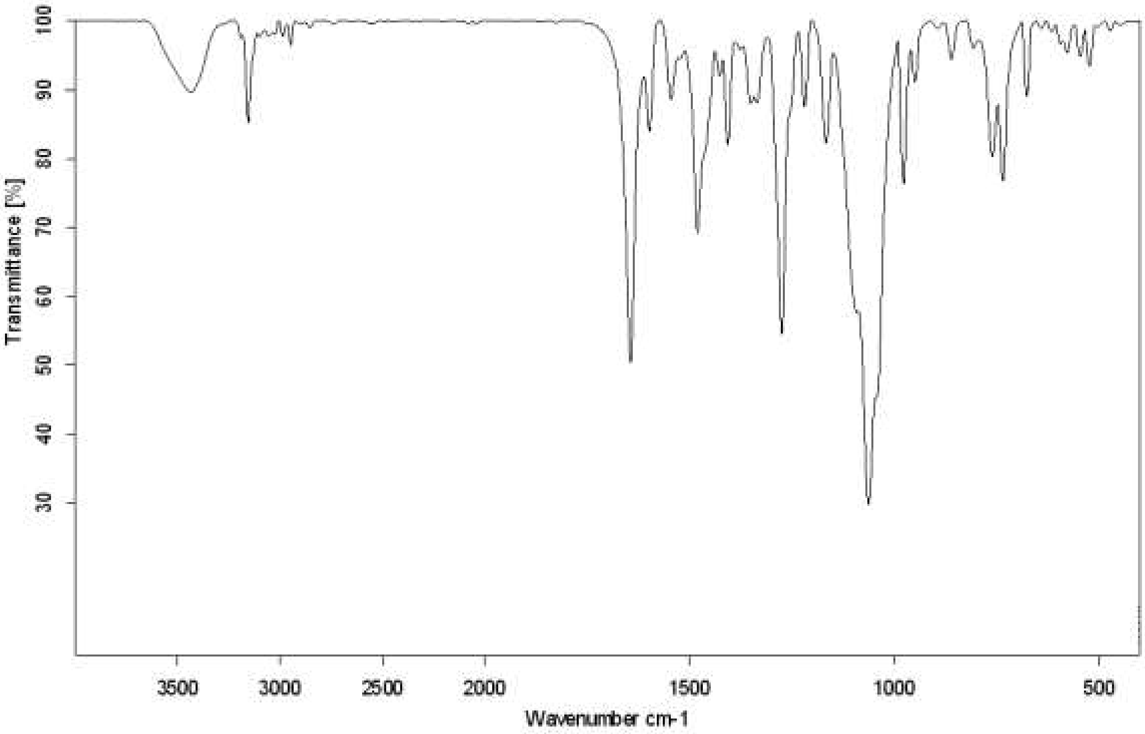

Fig. S1: FTIR spectrum of (**3**a)

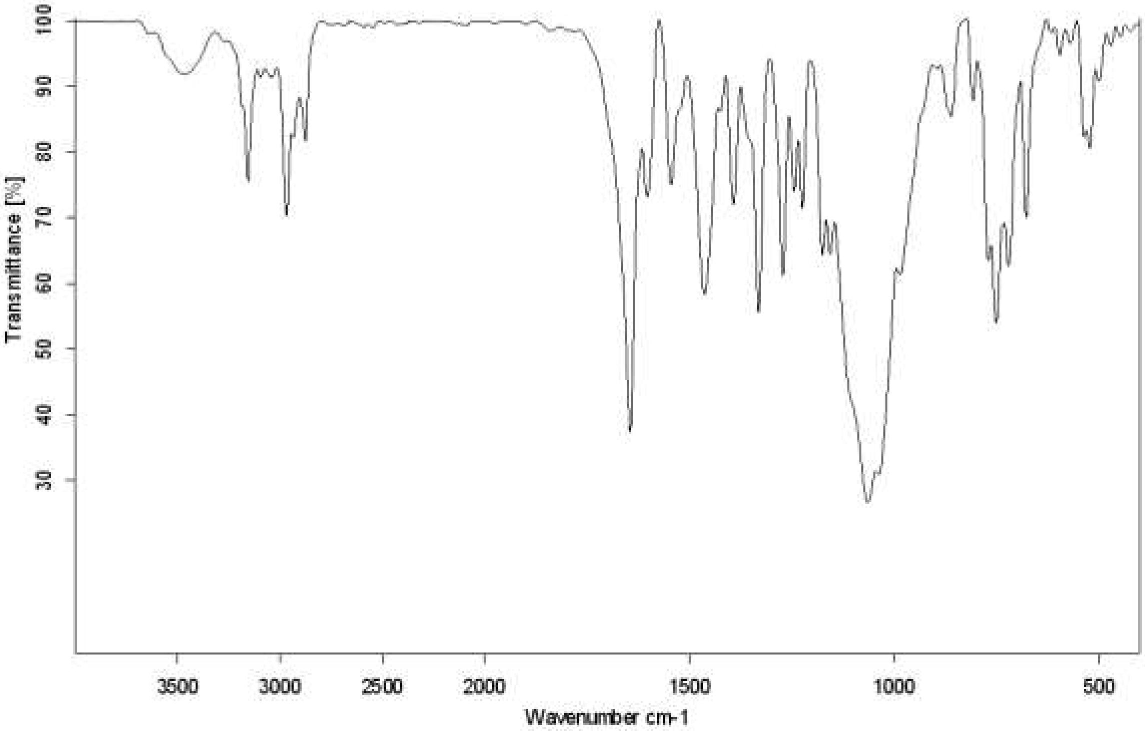

Fig. S2: FTIR spectrum of (**3**b)

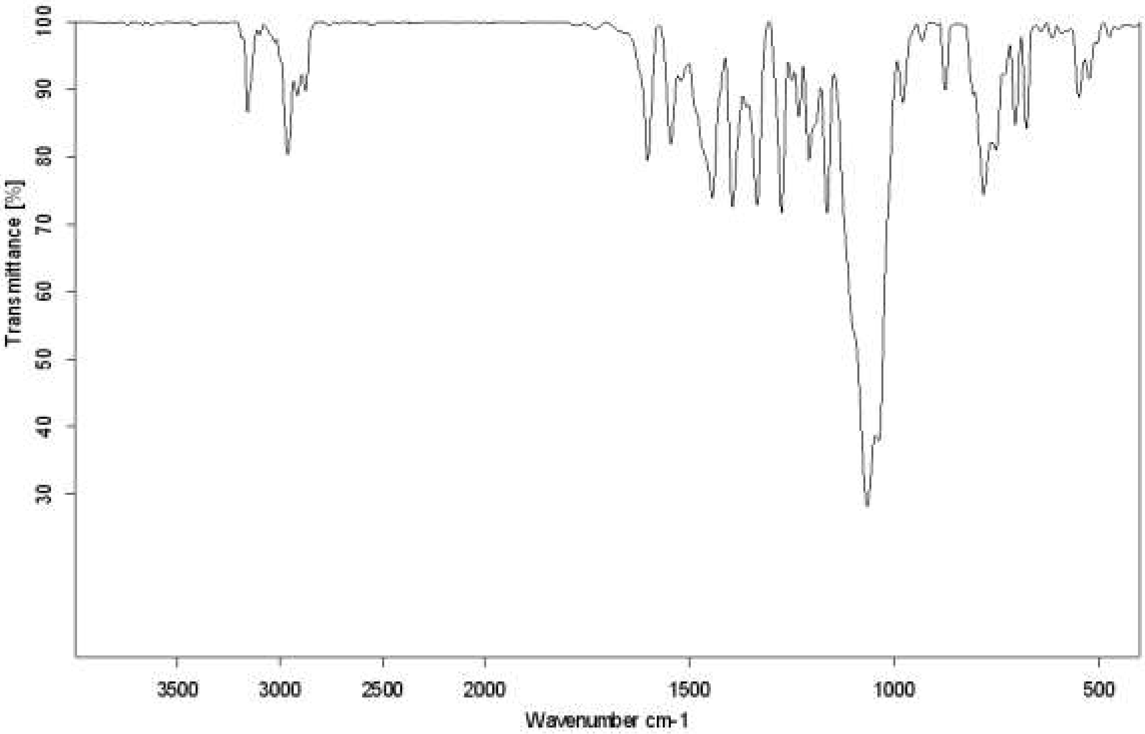

Fig. S3: FTIR spectrum of (**3**c)

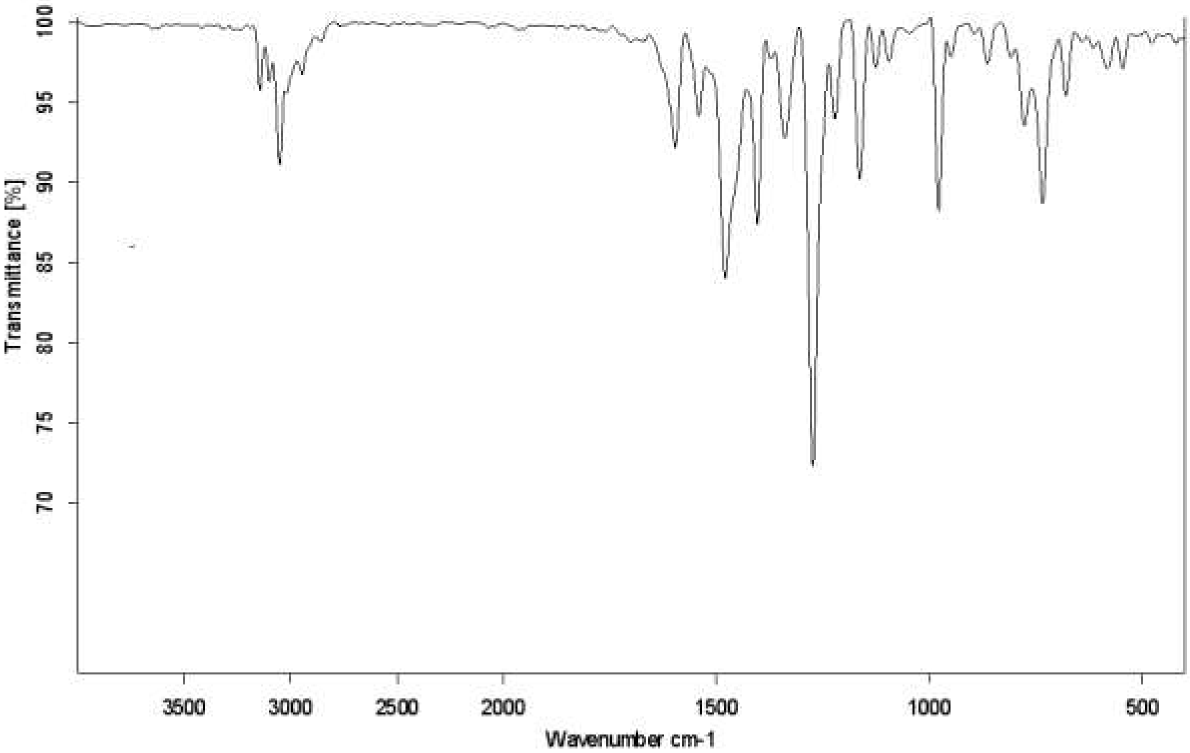

Fig. S4: FTIR spectrum of (**4**a)

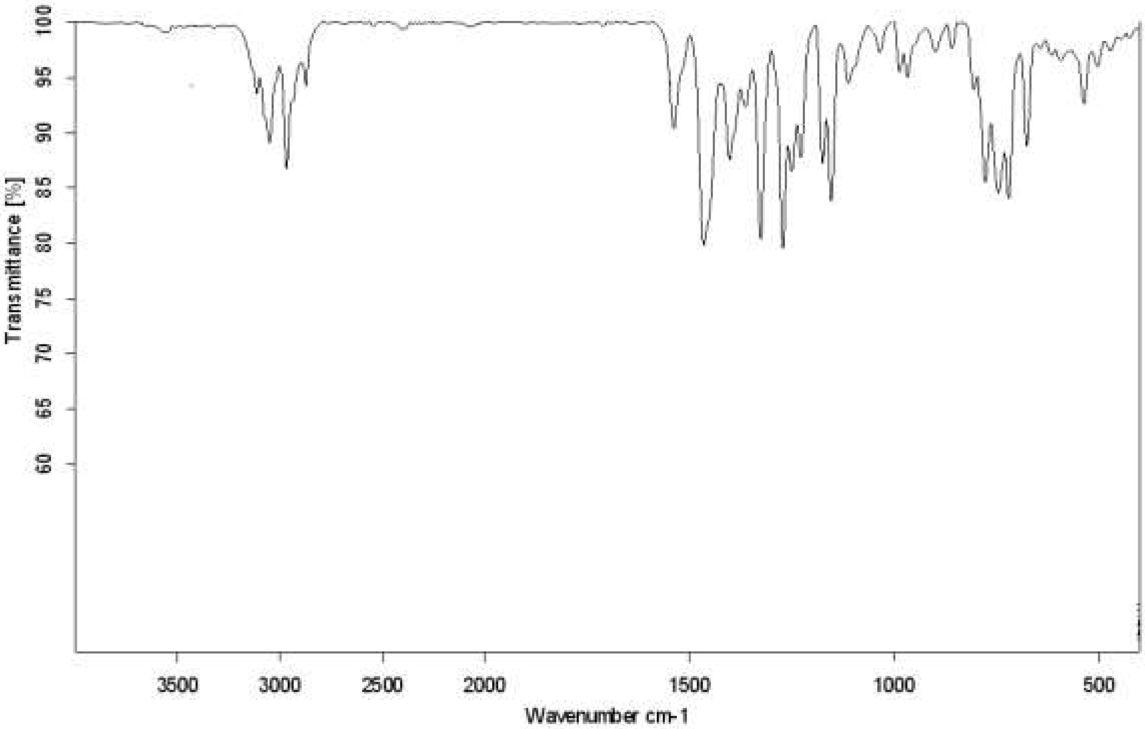

Fig. S5: FTIR spectrum of (**4**b)

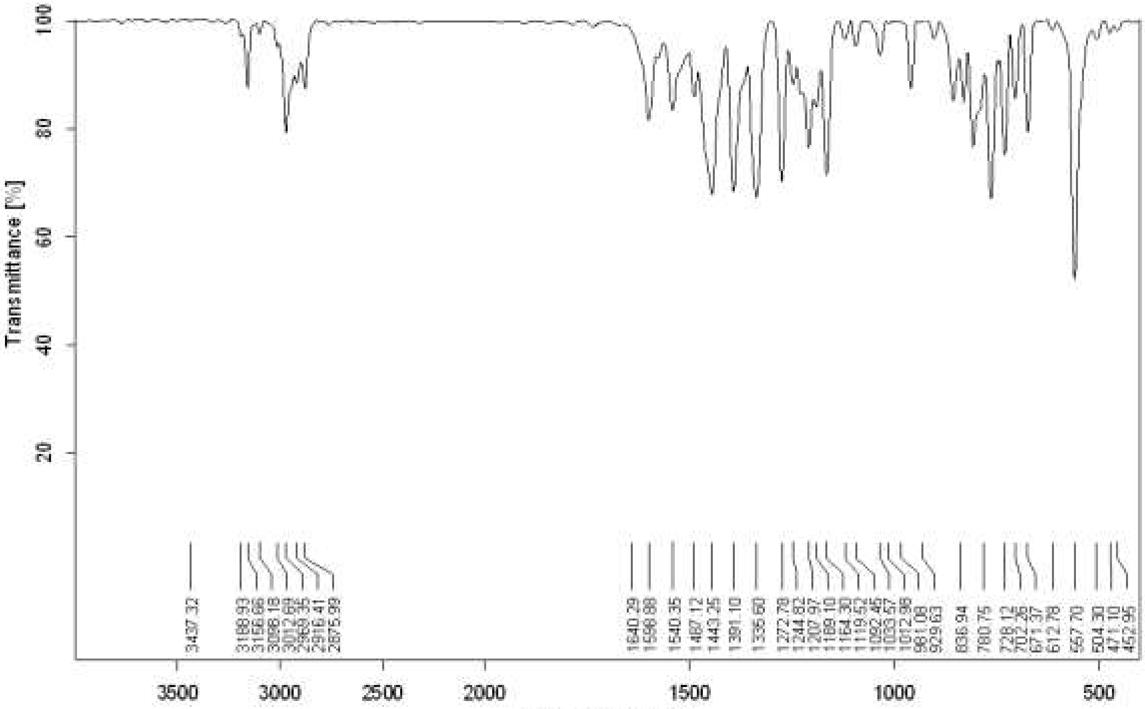

Fig. S6: FTIR spectrum of (**4**c)

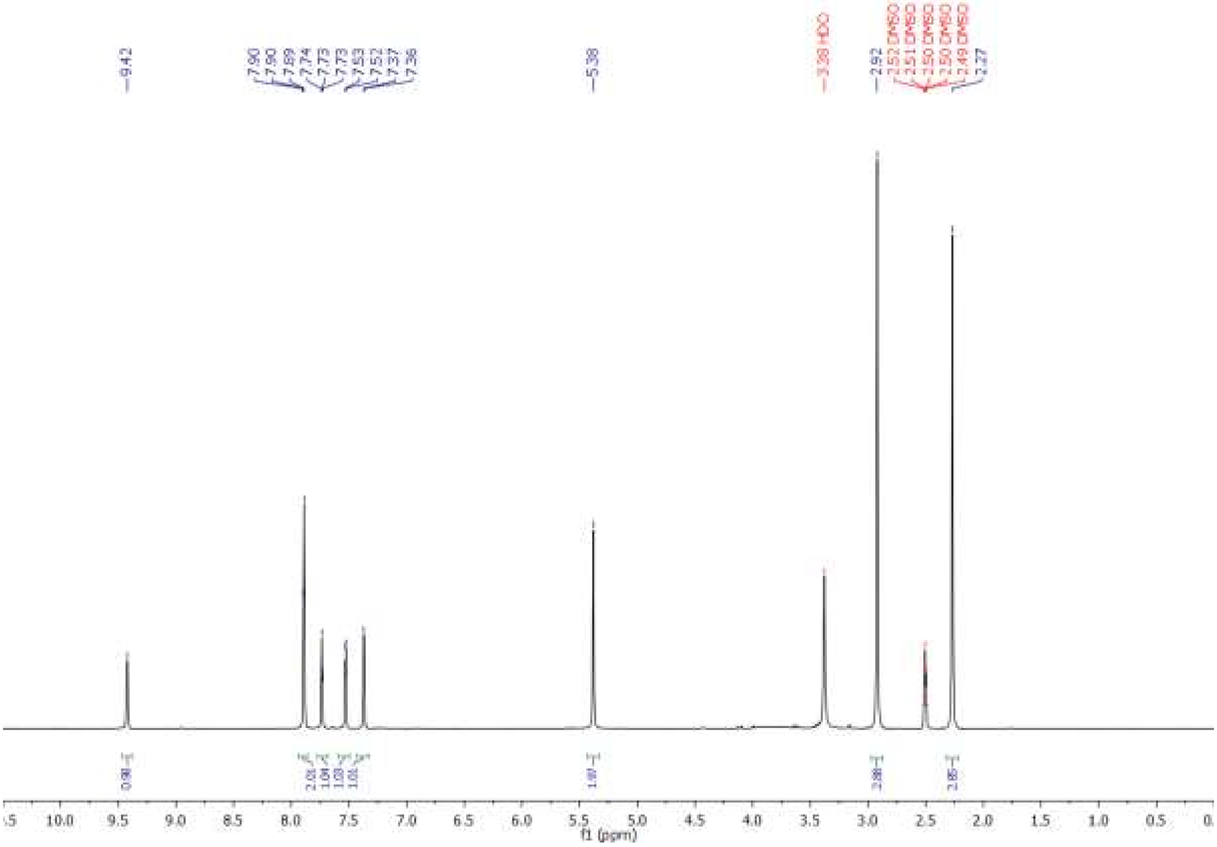

Fig. S7: ^{1}H NMR of **2a** (200 MHz, DMSO-d_6)

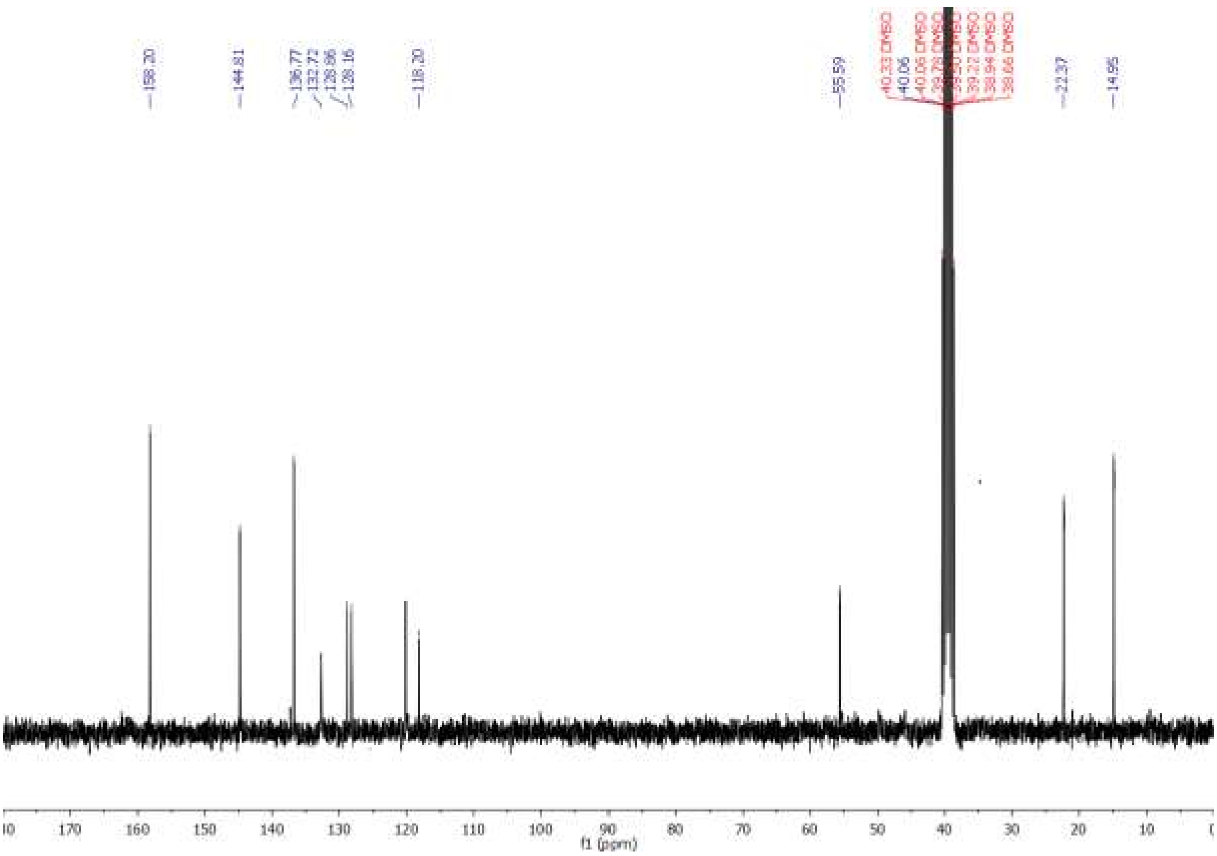

Fig. S8: ^{13}C NMR of **2a** (125 MHz, DMSO-d_6)

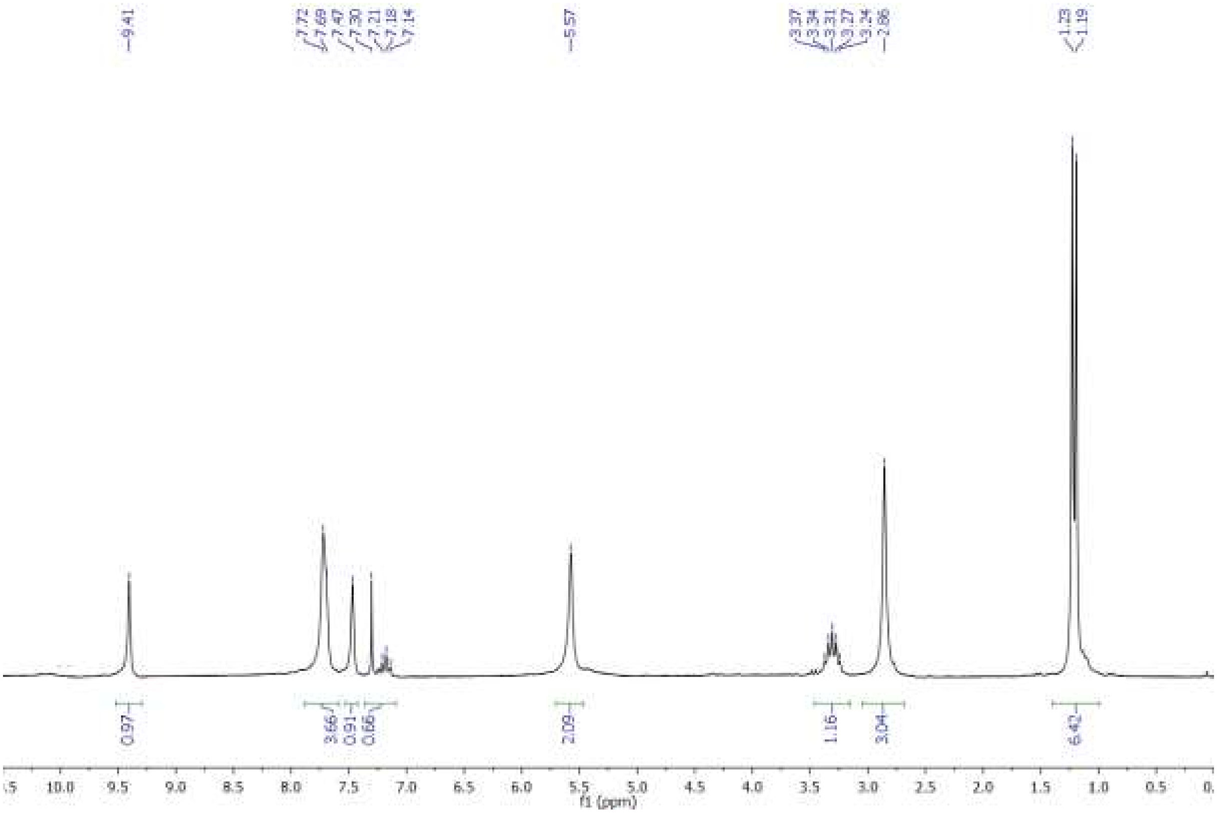

Fig. S9: ^{1}H NMR of **2b** (200 MHz, $CDCl_3$)

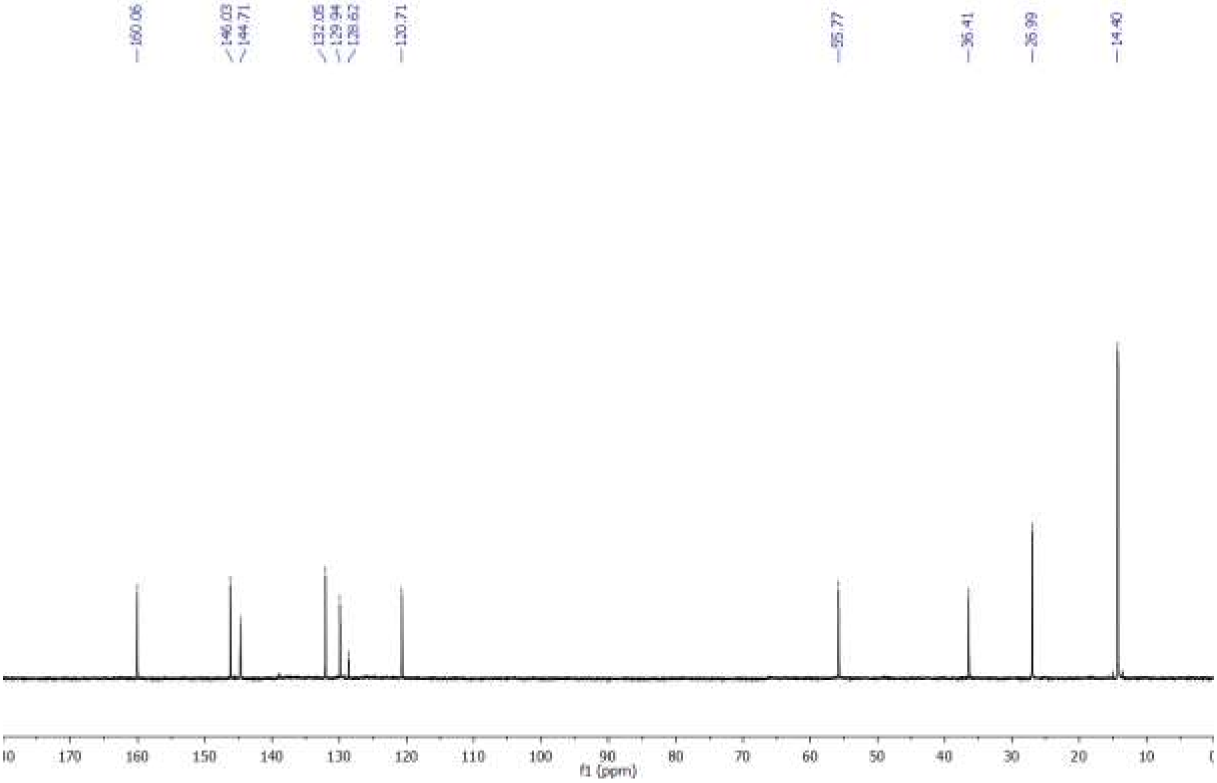

Fig. S10: ^{13}C NMR of **2b** (125 MHz, $CDCl_3$)

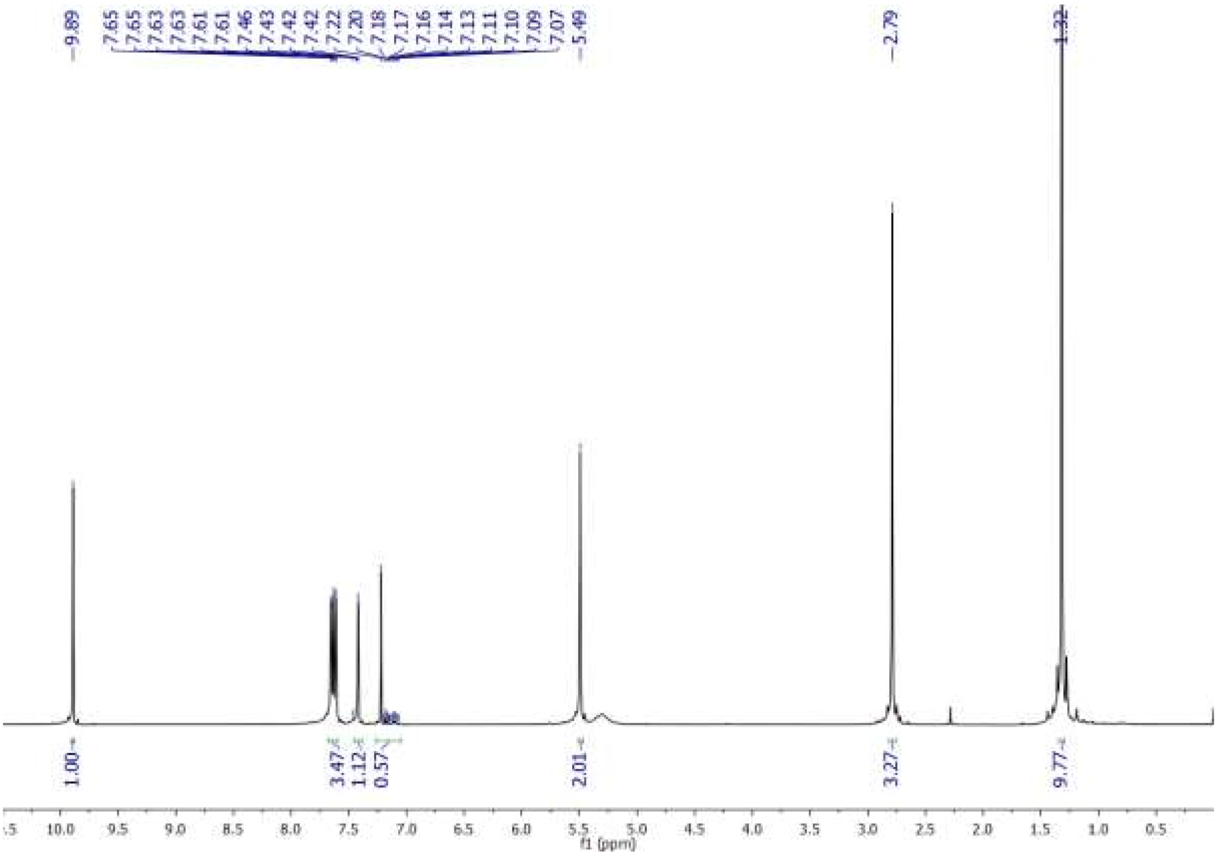

Fig. S11: ^{1}H NMR of **2c** (200 MHz, $CDCl_3$)

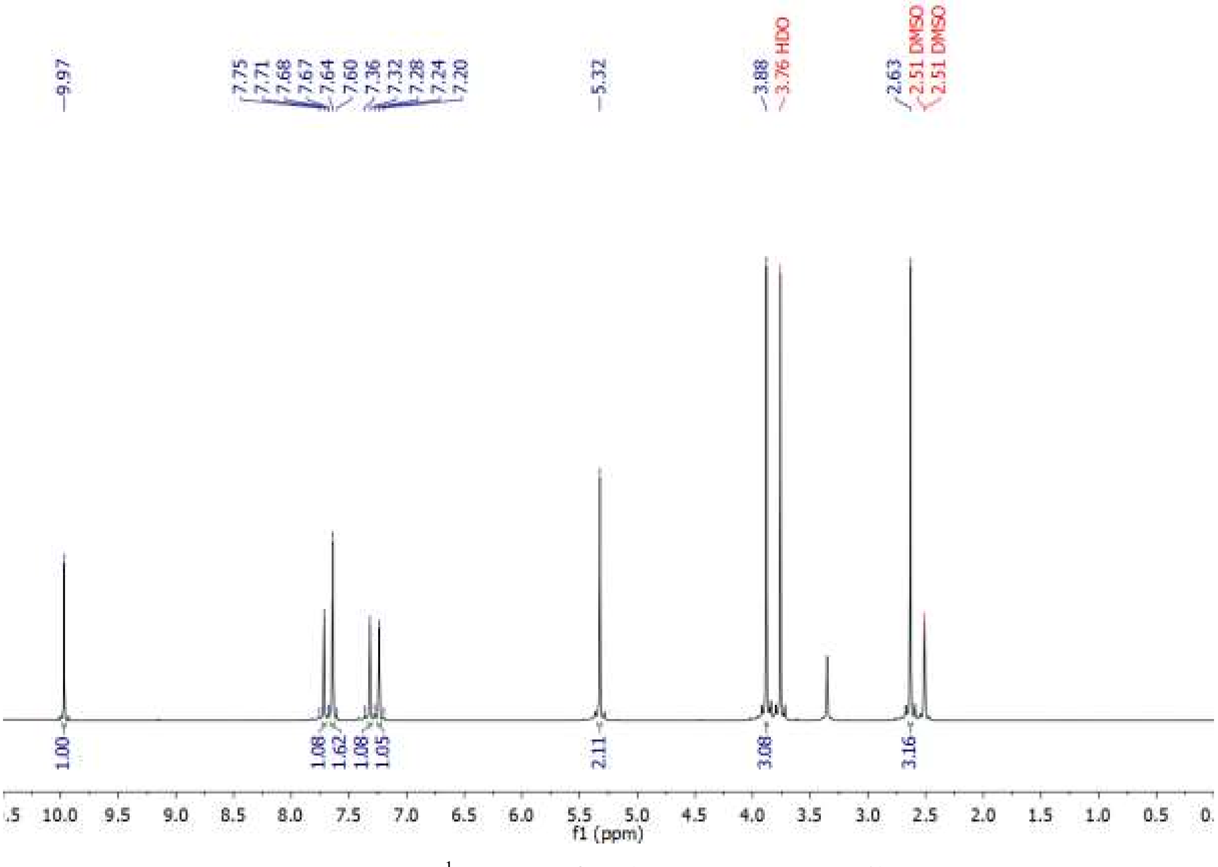

Fig. S12: ^{1}H NMR of **3a** (200 MHz, DMSO-d_6)

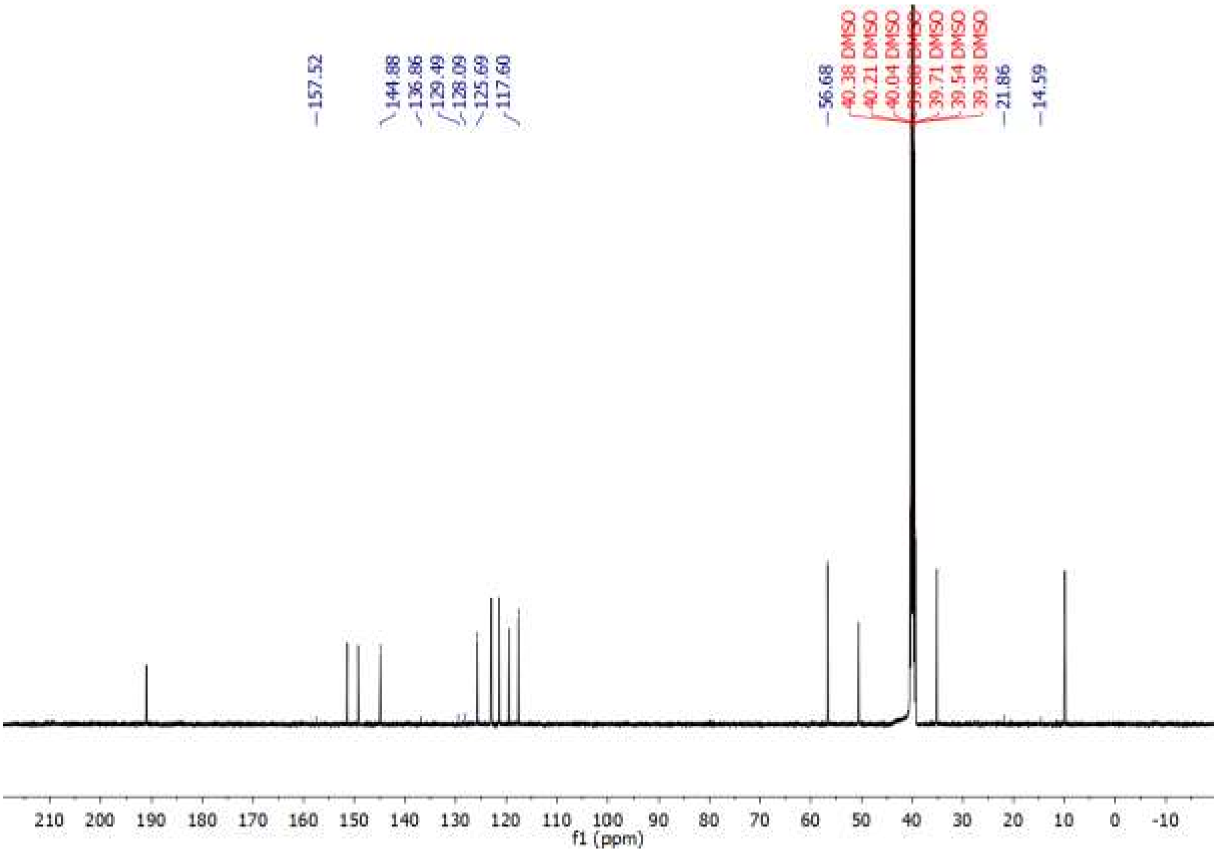

Fig. S13: ^{13}C NMR of **3a** (125 MHz, DMSO-d_6)

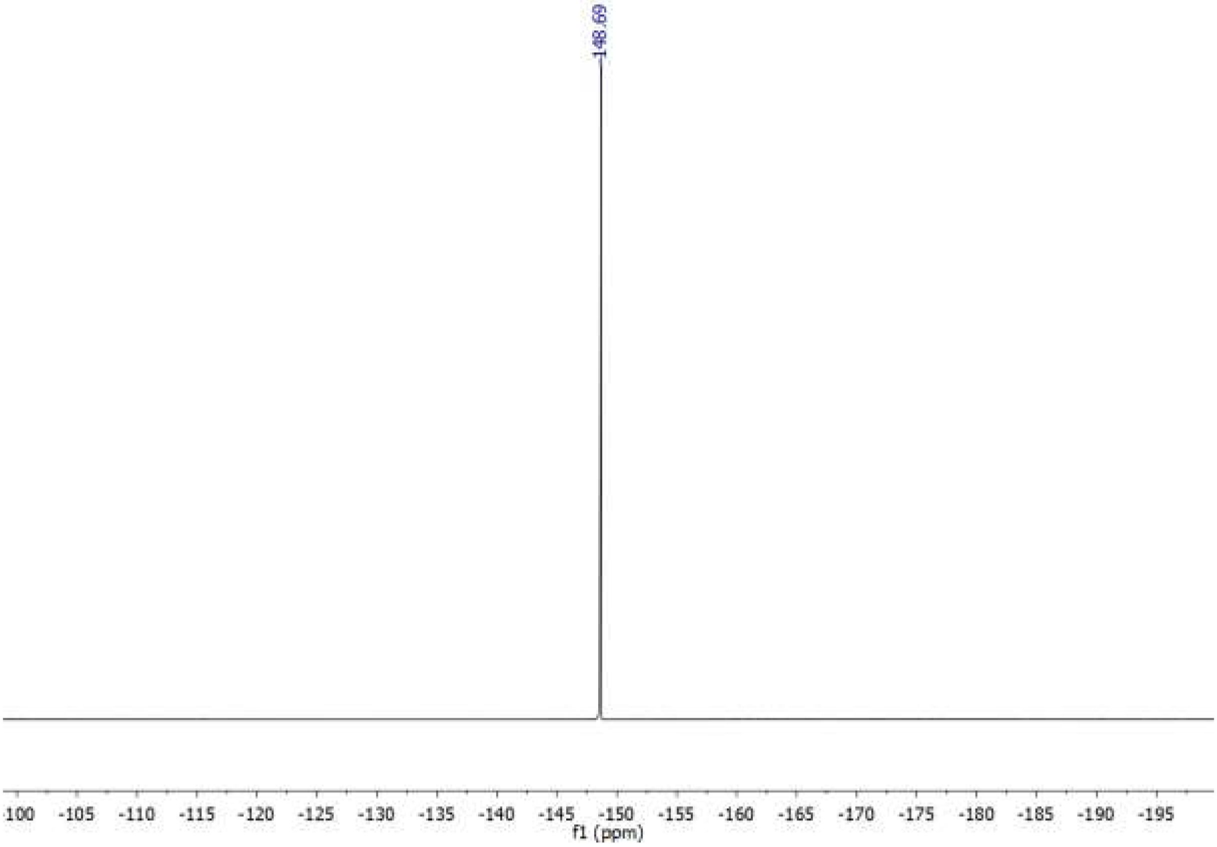

Fig. S14: ^{19}F NMR of **3a** (471 MHz, DMSO-d_6)

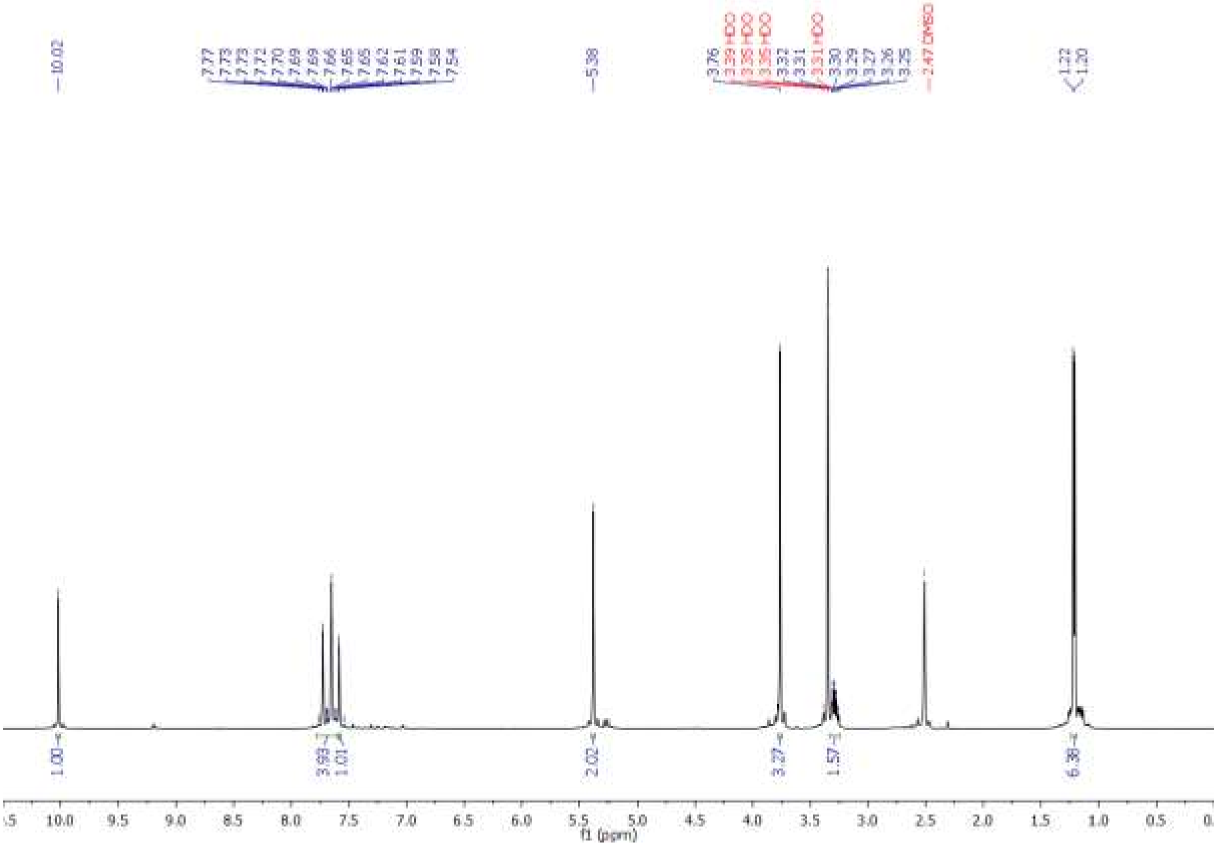

Fig. S15: ^{1}H NMR of **3b** (200 MHz, DMSO-d_6)

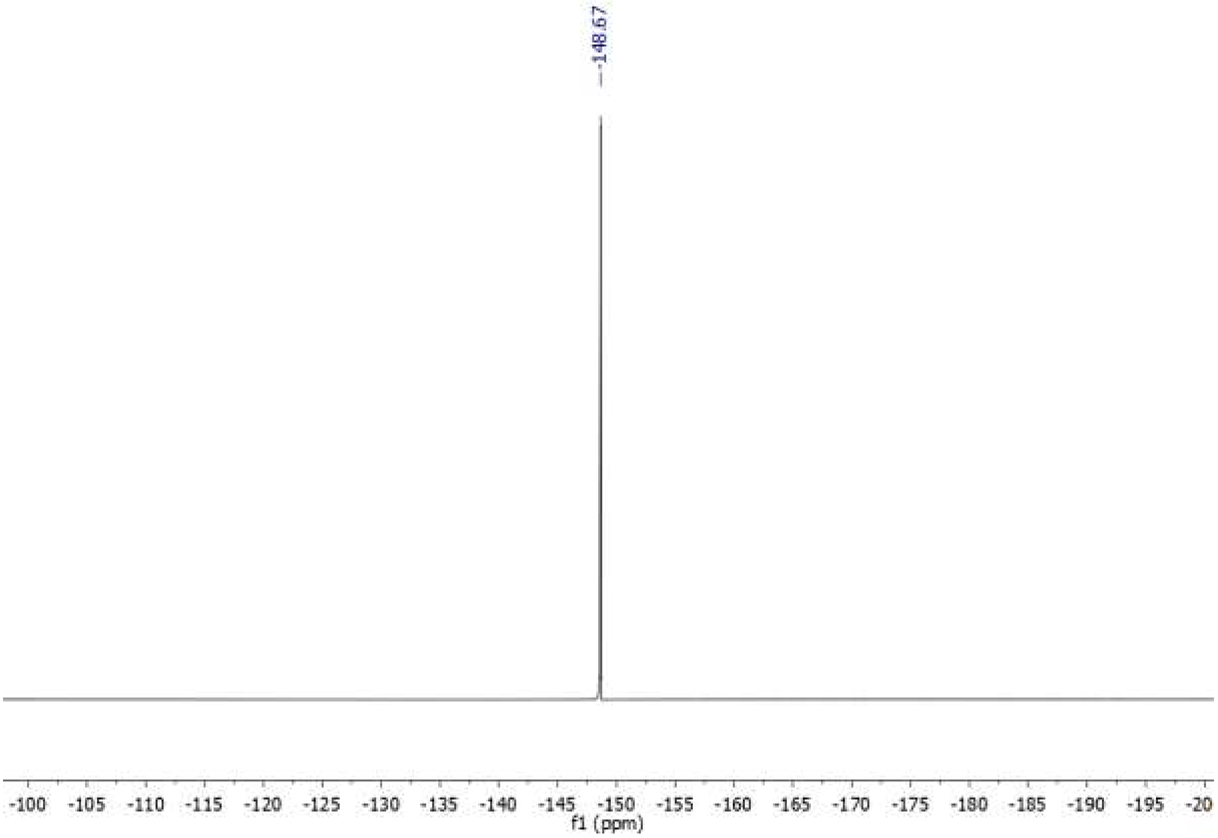

Fig. S16: ^{19}F NMR of **3b** (471 MHz, DMSO-d_6)

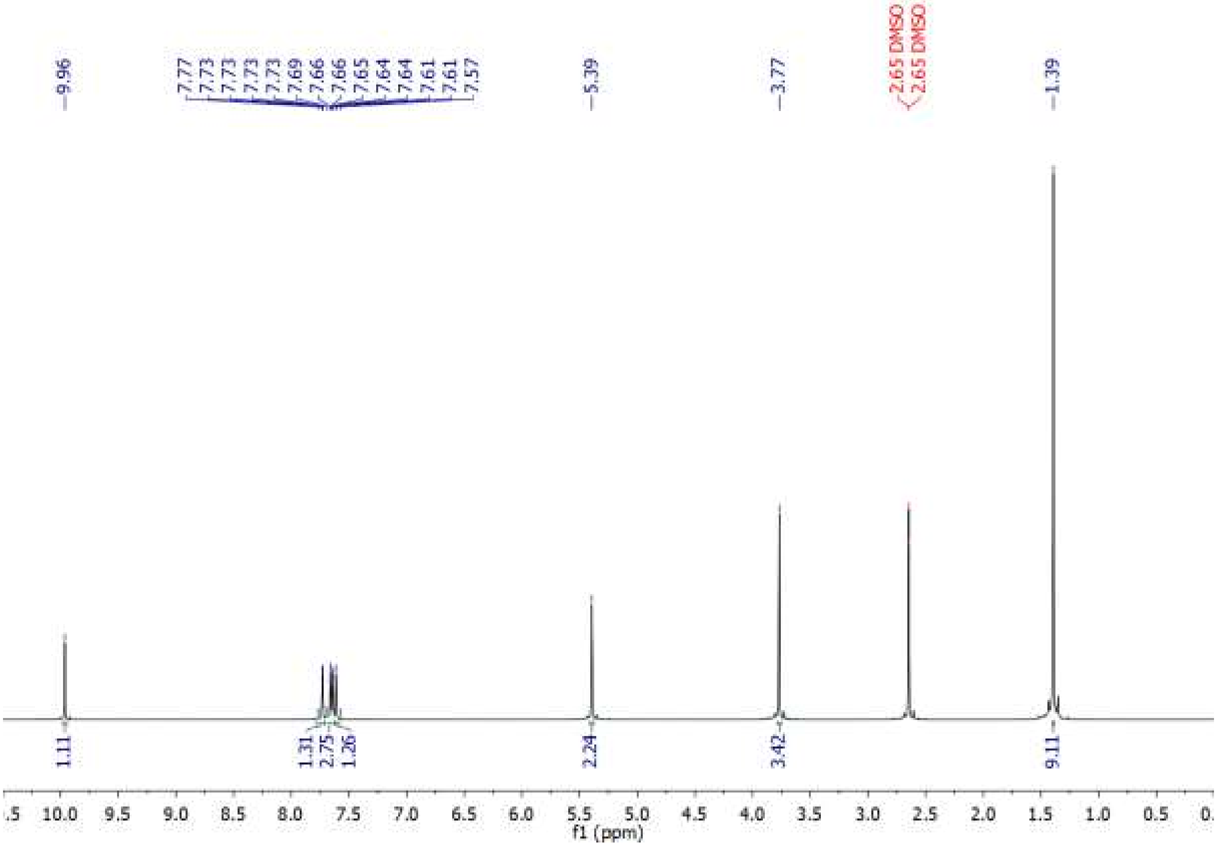

Fig. S17: ^{1}H NMR of **3c** (200 MHz, DMSO-d_6)

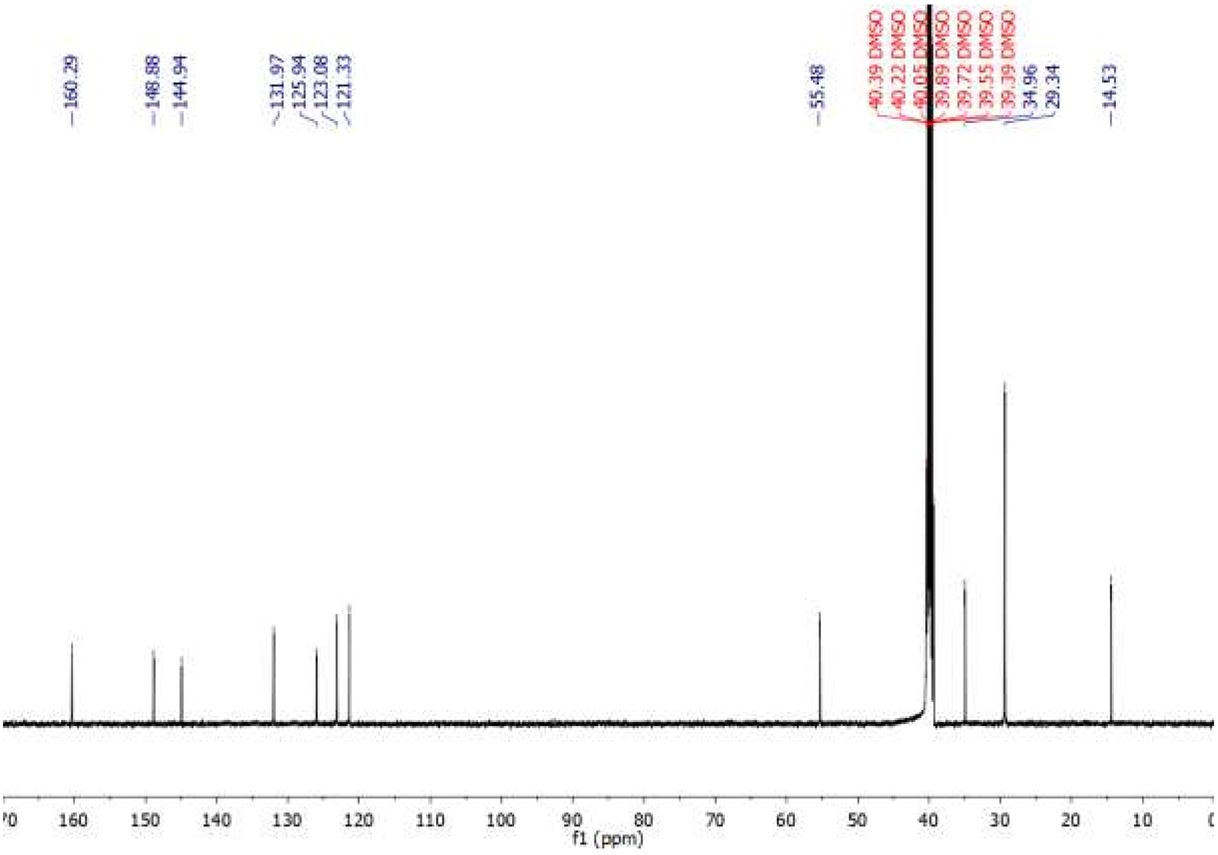

Fig. S18: ^{13}C NMR of **3c** (125 MHz, DMSO-d_6)

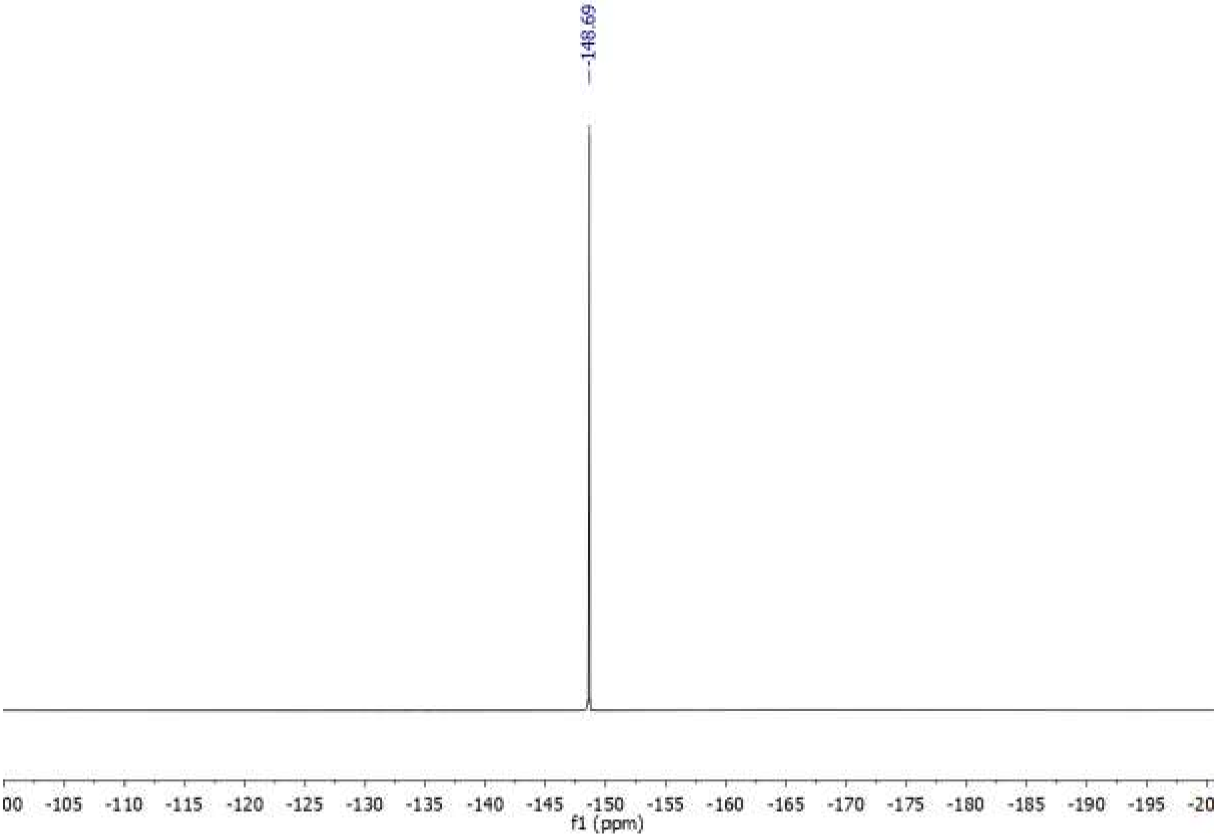

Fig. S19: ^{19}F NMR of **3c** (471 MHz, DMSO-d_6)

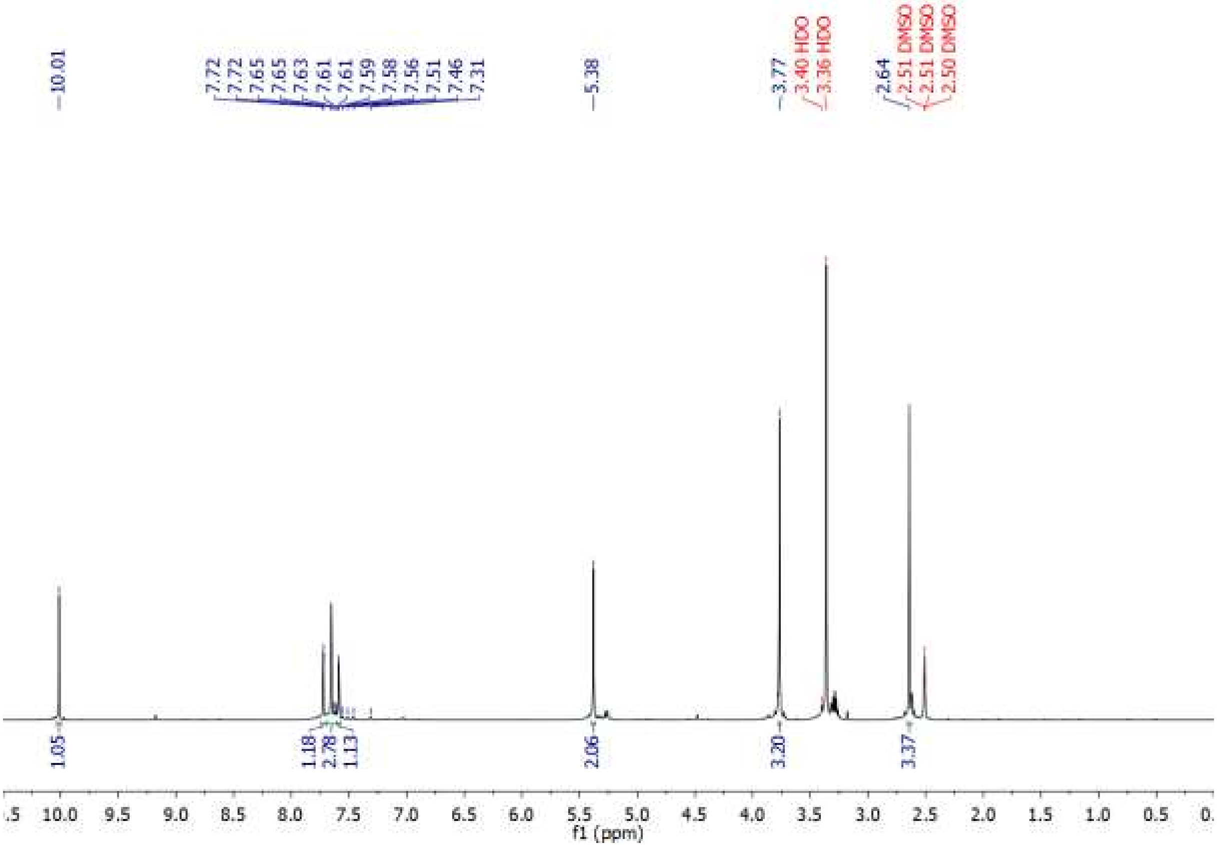

Fig. S20: ^{1}H NMR of **4a** (200 MHz, DMSO-d_6)

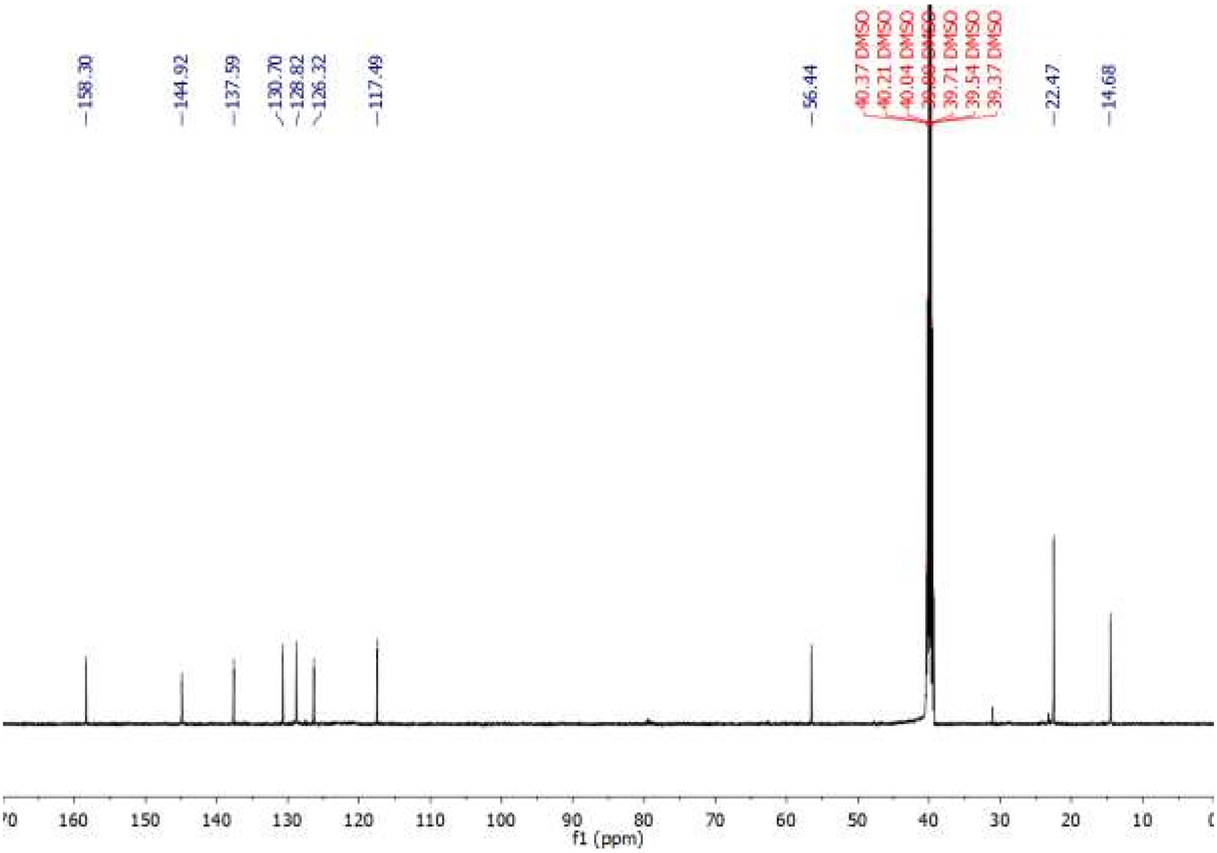

Fig. S21: ^{13}C NMR of **4a** (125 MHz, DMSO-d_6)

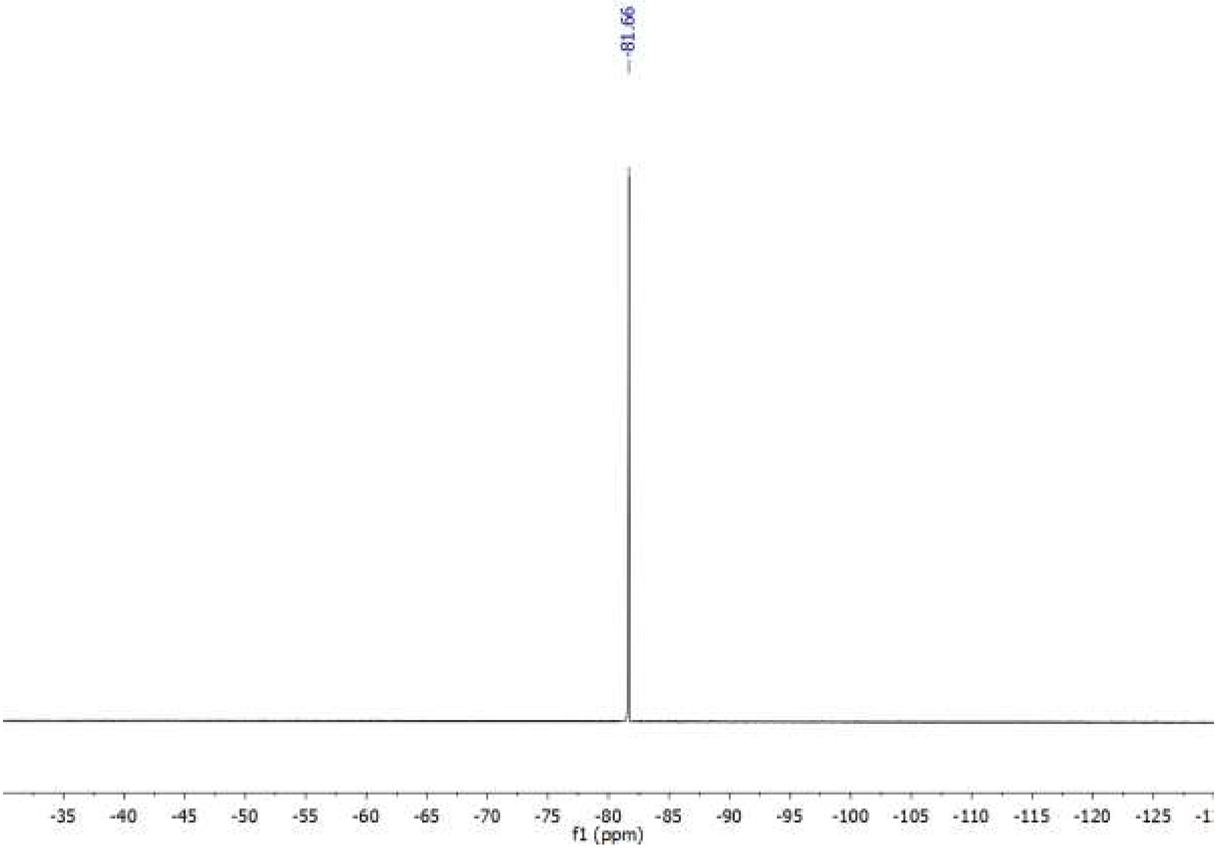

Fig. S22: ^{19}F NMR of **4a** (565 MHz, DMSO-d_6)

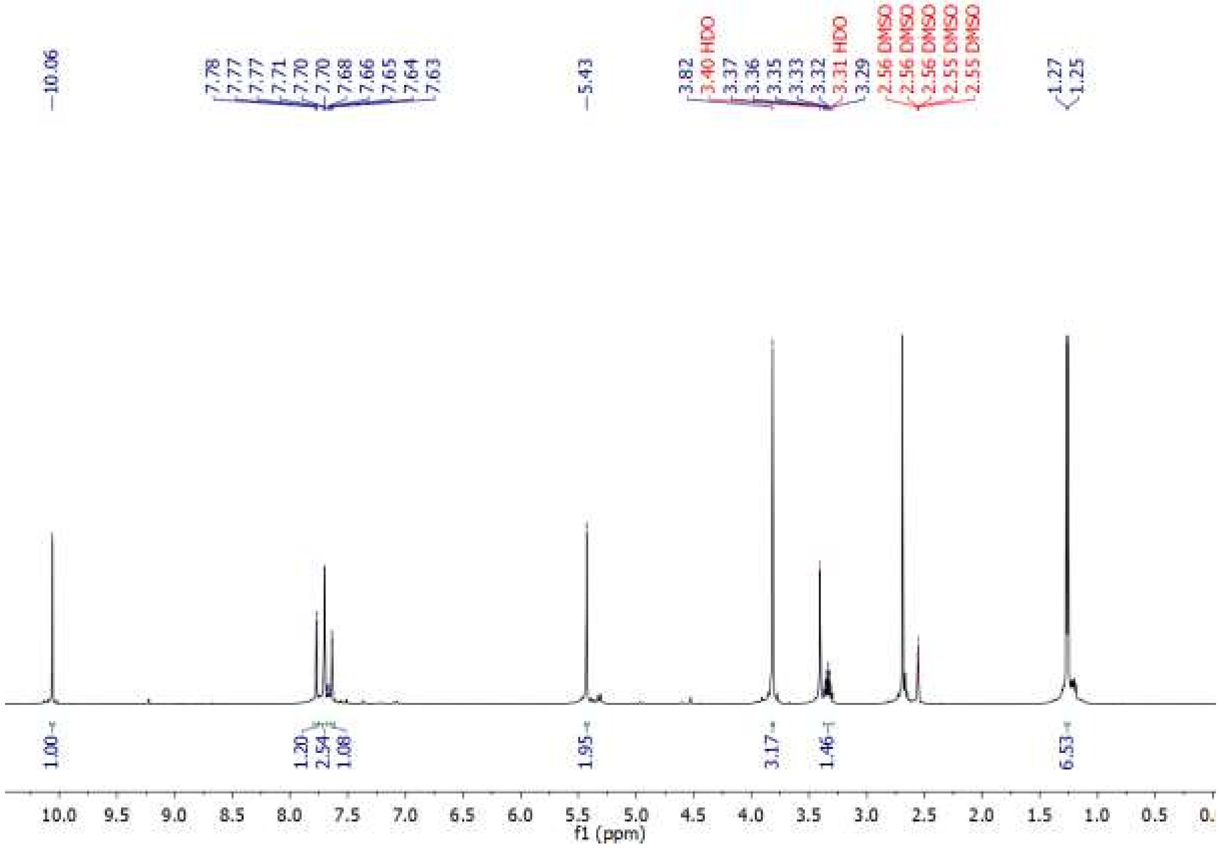

Fig. S23: ^{1}H NMR of **4b** (200 MHz, DMSO-d_6)

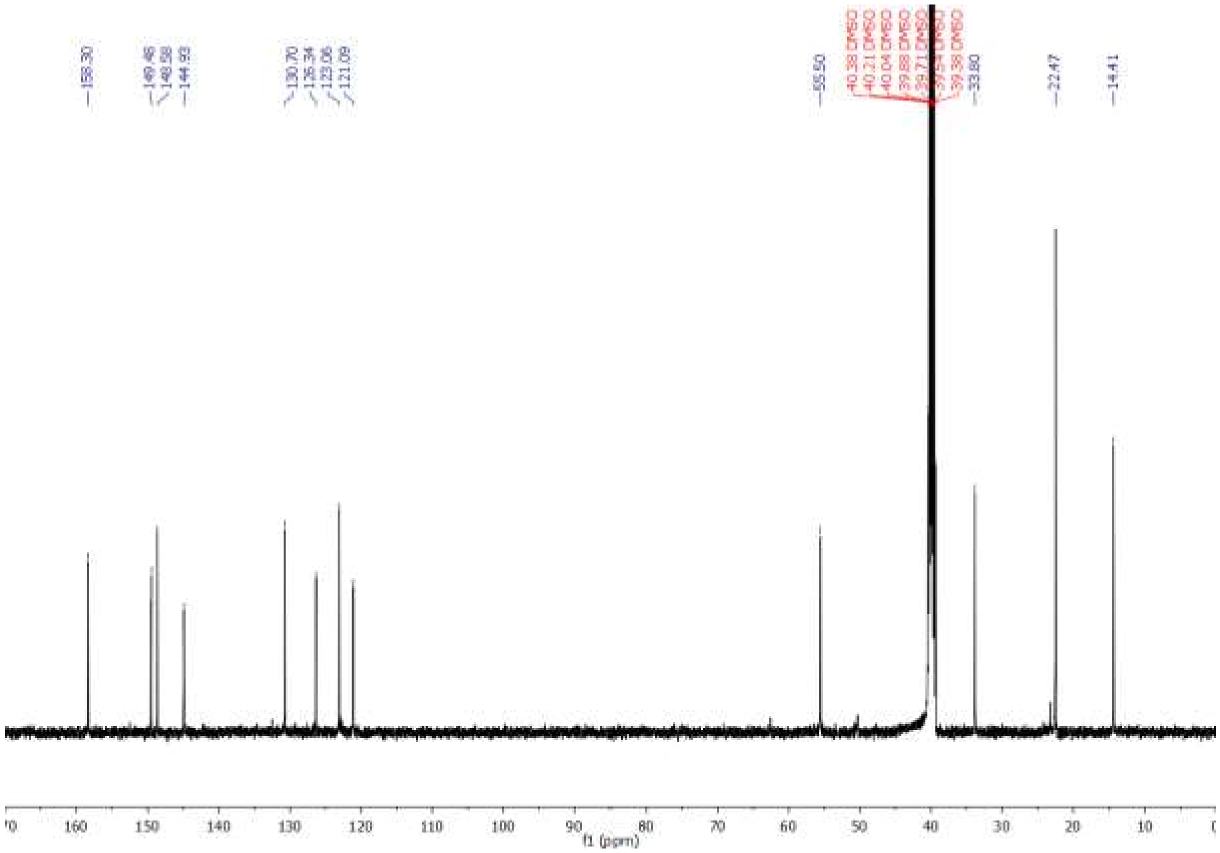

Fig. S24: ^{13}C NMR of **4b** (125 MHz, DMSO-d_6)

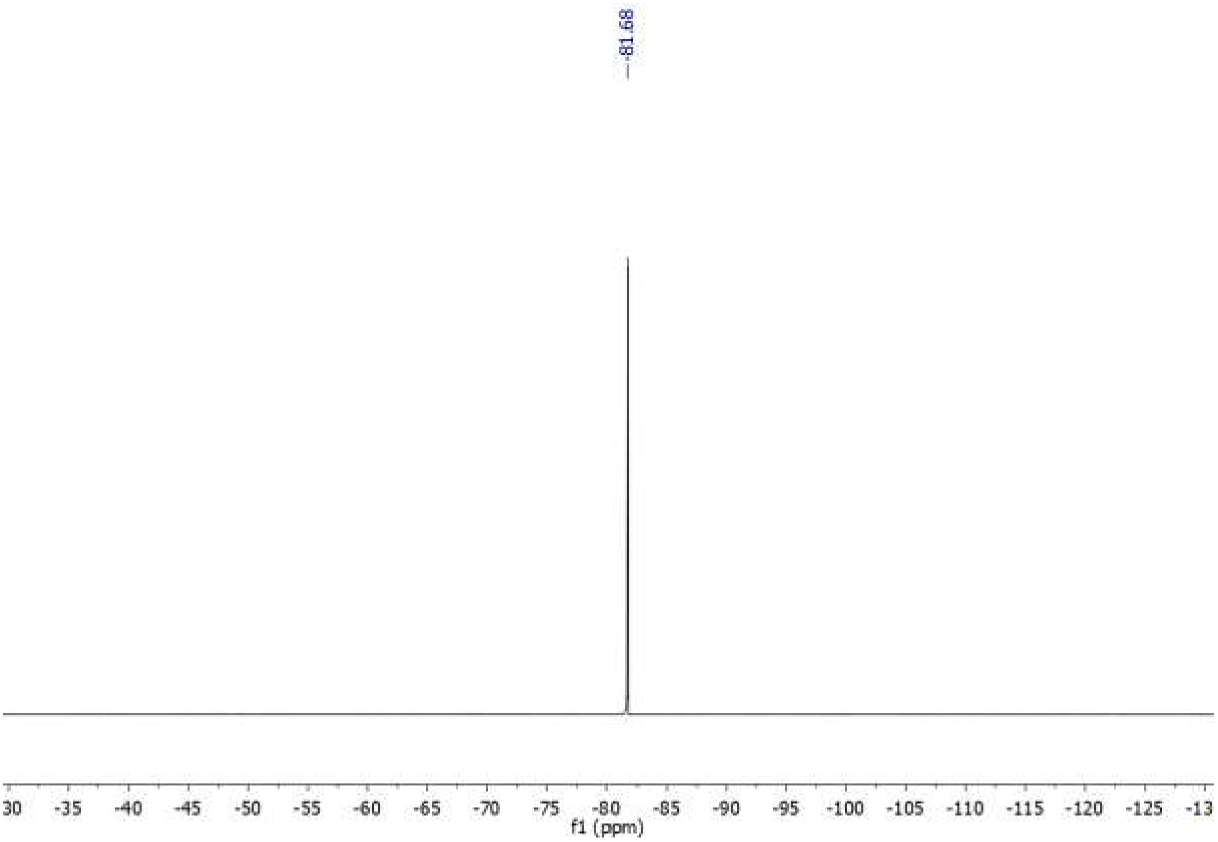

Fig. S25: ^{19}F NMR of **4b** (565 MHz, DMSO-d_6)

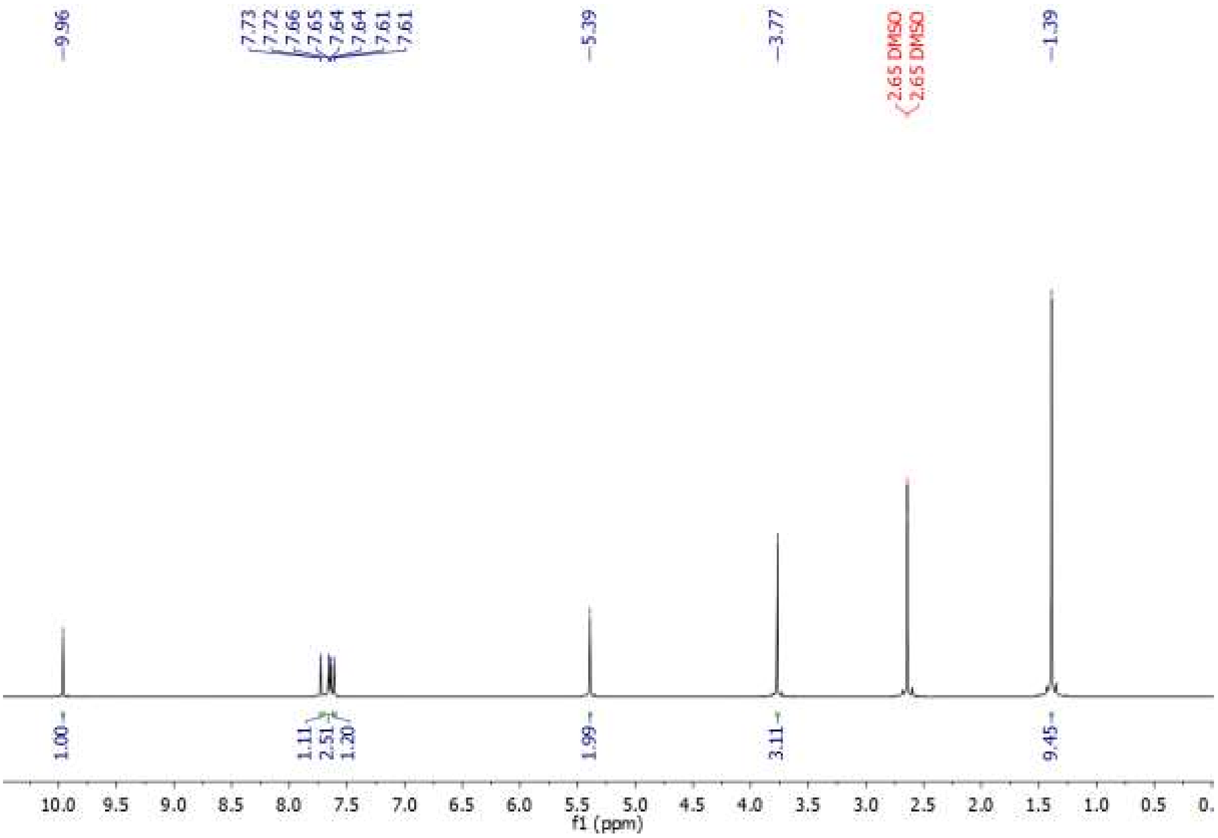

Fig. S26: ^{1}H NMR of **4c** (200 MHz, DMSO-d_6)

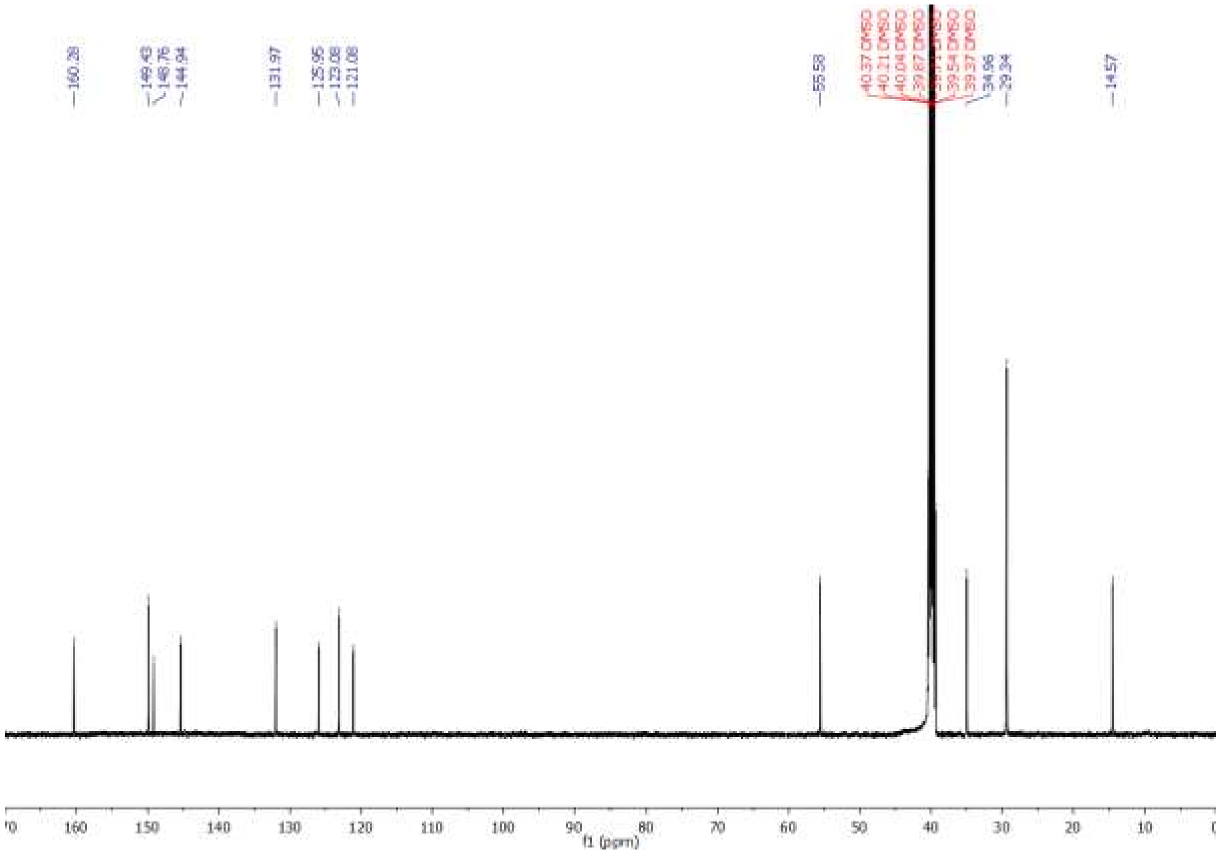

Fig. S27: ^{13}C NMR of **4c** (125 MHz, DMSO-d_6)

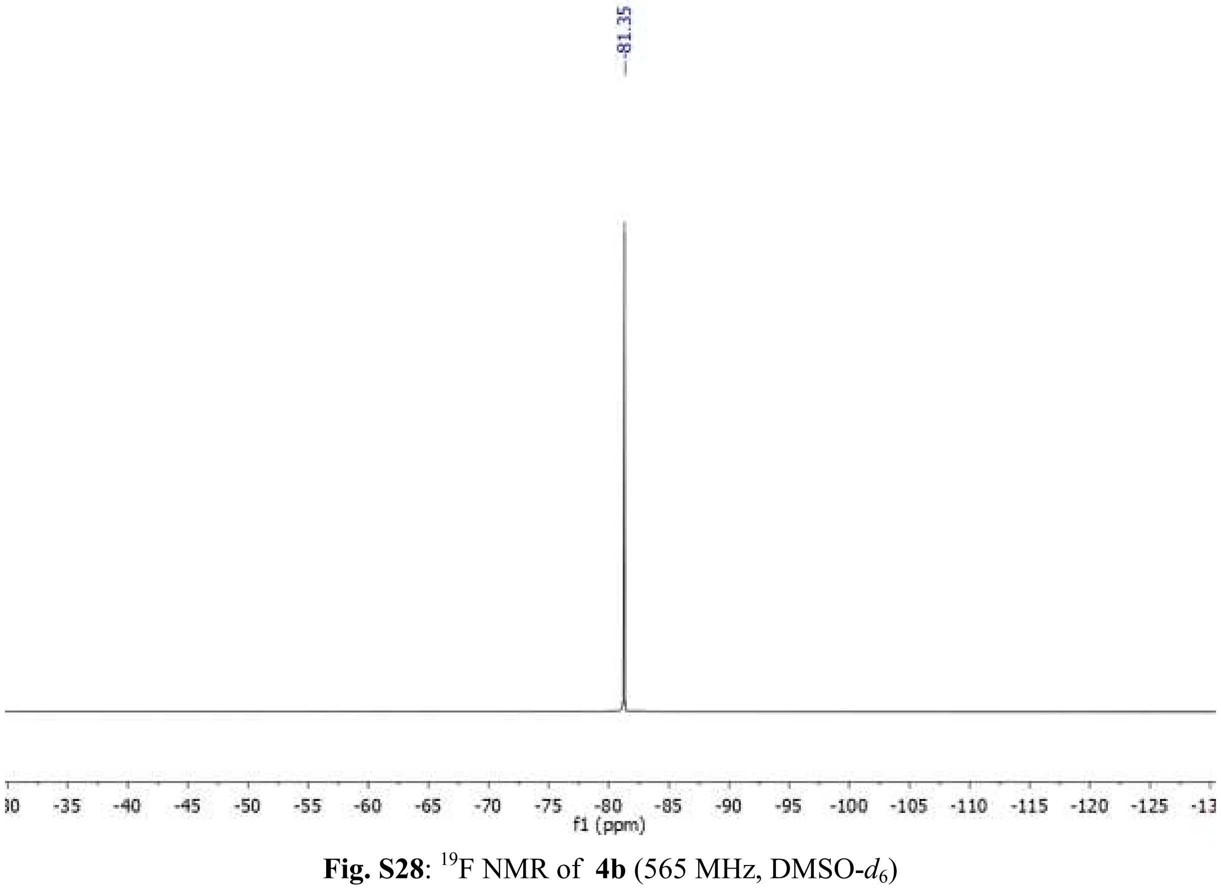

Fig. S28: ^{19}F NMR of **4b** (565 MHz, DMSO-d_6)

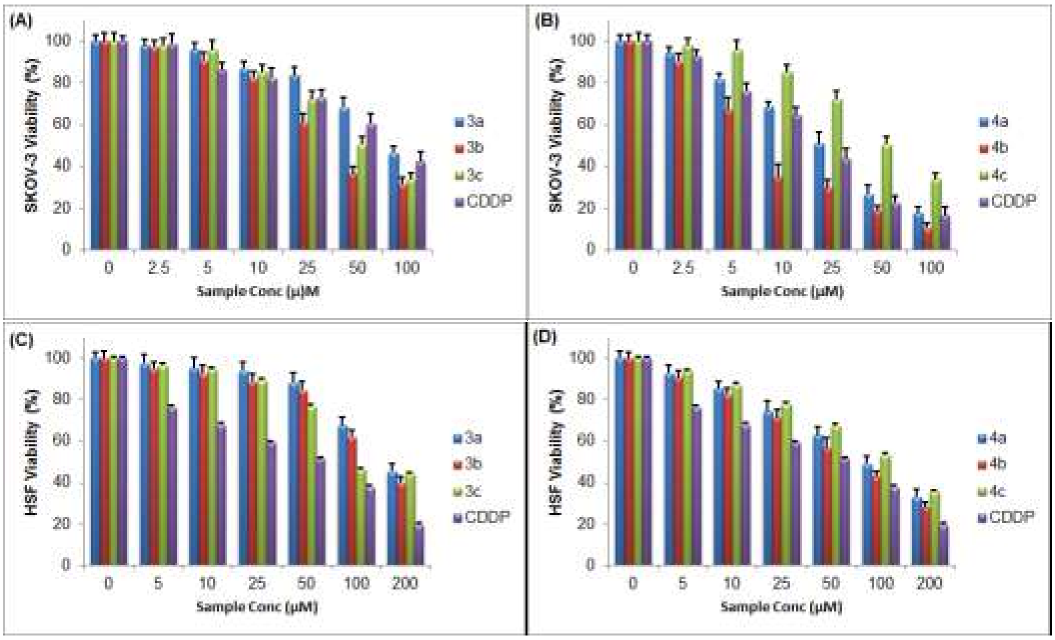

Fig. S29: The effect of different doses (0 – 200 µM) of TILs (**3a-c**) and (**4a-c**) on the viability of (**A,B**) ovarian carcinoma cell lines (SKOV-3) and (**C,D**) human skin fibroblast (HSF) cells after 24 h of treatment as compared to the clinical antitumor drug (cisplatin, CDDP).
